# Analysis of Changes in Taste Characteristics of Coffee at Different Primary Processing Methods Using E-Tongue, Untargeted Metabolomics and WGCNA

**DOI:** 10.3390/foods15091475

**Published:** 2026-04-23

**Authors:** Ying Liang, Yaqian Yuan, Jia Wang, Wenxue Chen, Weijun Chen, Qiuping Zhong, Jianfei Pei, Chun Chen, Xiong Fu, Rongrong He, Haiming Chen

**Affiliations:** 1 Hainan Nongken Investment Holding Group Co., Ltd., Collaborative Innovation Laboratory, College of Food Sciences & Engineering, Hainan University, 58 People Road, Haikou 570228, China; 2 School of Food Science and Engineering, South China University of Technology, Guangzhou 510640, China; 3 Haikou Key Laboratory of Special Foods, Haikou 570228, China

**Keywords:** Hainan Robusta coffee, primary processing methods, untargeted metabolomics, taste characteristics, weighted gene co-expression network analysis

## Abstract

The primary processing shapes the taste characteristics of coffee beans, while the regulation pathways remain unclear. Coffee beans processed by five methods—dry processing (DP), wet processing (WP), red honey (RH), black honey (BH) and anaerobic fermentation (AF)—were evaluated using electronic tongue analysis, sensory evaluation, and untargeted metabolomics. Sensory evaluation scores for mouthfeel, balance, and overall were higher in BH and AF. Conversely, the WP and DP exhibited heightened bitterness and astringency responses on the electronic tongue sensors, particularly for the former. The multigroup metabolomic comparison identified 808 DMs, and WGCNA revealed eight sensory-related modules containing 467 hub metabolites, mainly amino acids and derivatives, organic acids, alkaloids, and phenolic acids. KEGG analysis demonstrated that pathways such as caffeine metabolism and glycerophospholipid metabolism were the main pathways responsible for the metabolic differences. Further correlation analysis revealed potential flavor components closely associated with key taste characteristics. 1,3,4,5-tetrahydroxycyclohexanecarboxylic acid and Tyr demonstrated positive associations with bitterness, while TPC, TFC, Gly, and Met exhibited negative correlations with bitterness and astringency. Glu demonstrated a positive correlation with umami. These findings elucidate the material basis by which the primary processing modulates non-volatile compounds and taste perception, offering new insights into enhancing coffee quality.

## 1. Introduction

Coffee, a beverage with widespread global consumption, occupies a key position in global agricultural trade. With sustained economic growth and rising living standards, coffee has increasingly become a daily necessity that is closely associated with work, social interaction, and leisure activities [1]. The majority of the world’s primary coffee-producing regions are situated within the tropical belt, with Brazil, Vietnam, Colombia, and Ethiopia being among the foremost coffee-producing nations [2]. In China, coffee cultivation is concentrated primarily in Yunnan and Hainan. Among these, the province of Yunnan is predominantly characterized by the cultivation of *Coffea arabica* (Arabica coffee), while the tropical monsoon climate of Hainan offers more favorable conditions for the growth of *Coffea canephora* (Robusta coffee). The favorable ecological conditions of the island, characterized by abundant heat, high humidity, and volcanic red soils, create an ideal environment for Robusta cultivation. The ongoing expansion of the local coffee industry underscores the necessity for robust scientific evidence to support quality improvement.

The worldwide popularity of coffee is largely driven by its complex and pleasant flavor profile, which arises from the interaction between volatile aroma compounds and non-volatile taste-active components. A multitude of factors, including species and variety, terroir, harvest maturity, primary processing methods, storage conditions, roasting degree, and brewing parameters, collectively influence the final flavor profile of coffee [3]. Among these factors, primary processing, which involves the transformation of fresh coffee cherries into stable green coffee beans, plays a central role in determining the retention, degradation, and conversion of flavor precursors. Inappropriate processing has the potential to compromise intrinsic flavor characteristics or introduce fermentation defects, underscoring the importance of understanding how primary processing modulates the chemical basis of flavor and overall sensory quality.

Conventional primary processing methodologies encompass natural (dry), washed (wet), and honey processing techniques. In recent years, novel approaches such as anaerobic fermentation and carbonic maceration have been introduced [4]. The distinguishing characteristics of these processing pathways include variations in the extent of mucilage removal, the dynamics of water migration, and the intensity and composition of microbial activity. These variations result in distinct transformations in sugars, organic acids, phenolic compounds, lipids, peptides, and other nitrogenous substances. Consequently, coffees processed by different methods exhibit marked differences in acidity, bitterness, astringency, mouthfeel, and flavor complexity [5]. However, extant studies have predominantly focused on Arabica coffees, and systematic investigations of Robusta, particularly that cultivated in Hainan, China, remain limited. Additionally, the correlation between processing-induced metabolic changes and specific taste characteristics remains incompletely understood. Therefore, it is essential to elucidate the impact of diverse primary processing methods on the flavor quality of Robusta coffee of Hainan.

Untargeted metabolomics, a robust approach for comprehensively characterizing chemical variation without predefined targets, has been extensively applied to elucidate the impact of geographic origin, processing technology, roasting, and extraction conditions on the chemical composition and sensory properties of coffee [3]. In recent years, the development of network-based analytical frameworks, such as weighted gene co-expression network analysis (WGCNA), has facilitated the grouping of highly co-varying metabolites into modules and the correlation analysis of these modules to sensory traits. This advancement has enhanced the biological interpretability of complex metabolomics datasets [6,7]. Integration of UPLC-MS-based metabolomics with sensory evaluation and electronic tongue (E-tongue) analysis has enabled researchers to construct detailed chemical fingerprints and identify key metabolites associated with desirable sensory characteristics [8]. Therefore, the present study integrated E-tongue measurements, sensory evaluation, and untargeted UPLC-MS metabolomics to investigate the effects of different primary processing methods on the non-volatile metabolite profiles and taste characteristics of Hainan Robusta coffee. Multivariate statistical analysis and WGCNA are employed to identify metabolite modules and potential flavor markers associated with key taste characteristics. This study will provide theoretical foundations for elucidating the flavor mechanisms and quality enhancement of Robusta coffee of Hainan.

## 2. Materials and Methods

### 2.1. Chemicals and Reagents

Analytical-grade anhydrous ethanol, anhydrous methanol, Folin–Ciocalteu’s phenol, and anhydrous sodium carbonate were procured from Xilong Scientific Co., Ltd. (Shenzhen, China). HPLC-grade methanol, acetonitrile, and formic acid were obtained from Merck (Darmstadt, Germany) and Aladdin Biotech Co., Ltd. (Shanghai, China). Standards of gallic acid, rutin, caffeine, trigonelline, chlorogenic acid, oxalic acid, tartaric acid, quinic acid, malic acid, acetic acid, citric acid, and succinic acid (≥98%; HPLC) were supplied by Yuanye Bio-Technology Co., Ltd. (Shanghai, China).

### 2.2. Sample Priming and Roasting

#### 2.2.1. Primary Processing of Coffee Samples

Robusta coffee beans (DaFeng No. 1 cultivar) were harvested at the Mushan Coffee Factory in Qiongzhong Li and Miao Autonomous County, Hainan, China. During the harvesting process, coffee cherries that were both uniformly red and consistent in size were meticulously selected. Subsequently, the cherries were sorted to remove impurities and defective beans. Subsequently, the cherries were divided into five batches and subjected to various primary processing methods.

The dry process (DP) entails the uniform distribution of cherries and their subsequent natural drying under sunlight. In the washed process (WP), the cherries underwent a sequence of steps that included depulping, followed by a washing procedure that was designed to remove mucilage, and subsequently, sun-drying. The red honey process (RH) necessitated the depulping of cherries and the extraction of a portion of the mucilage, while ensuring the retention of 50–60% of the original mucilage. The beans were then subjected to a fermentation process under constant temperature and humidity conditions (25 °C, 75% humidity) for a duration of 15 days. This process was undertaken until the mucilage layer was completely removed. Subsequent to this, the beans underwent a natural drying process through sun exposure. The black honey process (BH) entails the extraction of the pulp and a portion of the mucilage, resulting in the retention of 90–100% of the mucilage. The beans were subjected to a constant temperature and humidity (25 °C, 75% humidity) for a 22-day fermentation period, followed by a sun-drying process. The anaerobic fermentation process (AF) entailed the placement of depulped beans into sealed containers, thereby establishing an oxygen-free environment. These beans were then subjected to a 12-day fermentation process, followed by a natural sun-drying period. The moisture content of all treatment samples was reduced to 11.0 ± 1.0 g/100 g dry weight, as determined according to the Chinese Agricultural Industry Standard NY/T 604-2020 [9], with the objective of preventing quality degradation. Subsequently, the beans were processed to remove their outer layers, resulting in the extraction of green coffee beans for subsequent analysis.

#### 2.2.2. Coffee Sample Roasting Process

For the roasting process, the green coffee beans (500.00 g) were roasted in a 0.60 kg-capacity Bideli drum roaster (Bider General Equipment Co., Ltd., Guangzhou, China).

Roasting was conducted at an initial (charge) temperature of 190 °C, with a final temperature of approximately 210 °C and a total roasting time of 11–13 min, which corresponded to a medium roast. After roasting, the beans were ground using an AKIRAKOKI M-520A coffee grinder (Akira International Co., Ltd., Taiwan, China) and sifted through a standard sieve to remove larger particles.

### 2.3. E-Tongue-Assisted Taste Assessment

Taste attributes of coffee samples produced by five primary processing methods were evaluated using an electronic tongue system (SA402B, Smart Sensor Technology, Tokyo, Japan) according to a previously reported protocol [8]. Coffee grounds (0.50 g) were brewed with deionized water (50 mL) under boiling conditions for 5 min, after which the extracts were cooled and filtered prior to analysis. The assessment of umami, sourness, saltiness, bitterness, and astringency was conducted using the designated probes (AAE, CA0, CT0, C00, and AE1). Additionally, aftertaste-related parameters (aftertaste-B and aftertaste-A) and richness were obtained. Prior to the execution of measurements under controlled conditions (25 °C), the instrument underwent a thorough cleaning and calibration process. All measurements were conducted in triplicate, and sensor responses collected over 30 s were used for data analysis.

### 2.4. Determination of Total Phenols and Total Flavonoids

#### 2.4.1. Determination of Total Phenolic Content (TPC)

The quantification of TPC was performed using the Folin–Ciocalteu assay, as previously described [8]. Ground coffee (0.50 g) was subjected to ultrasonic extraction with 70% (*v*/*v*) methanol (25 mL) at ambient temperature. The extracts were subsequently subjected to centrifugation, and the resulting supernants from three independent extractions were then combined. For the colorimetric determination, an aliquot of the extract or gallic acid standard was permitted to react with Folin–Ciocalteu reagent and sodium carbonate for a period of two hours in a 96-well plate under conditions of darkness. The degree of absorption was measured at a wavelength of 765 nm by means of a microplate reader (SpectraMax iD3, Molecular Devices, San Jose, CA, USA). The results were reported as milligrams (mg) of gallic acid equivalents (GAE) per gram of dry weight (mg GAE/g DW).

#### 2.4.2. Determination of Total Flavonoid Content (TFC)

TFC was measured using the aluminum-chloride colorimetric method [10], with the same extract as for TPC determination. Rutin was utilized as the reference standard. For the assay, the extract or standard was sequentially reacted with ethanol, sodium nitrite, aluminum nitrate, and sodium hydroxide in a microplate format to allow color development. Following the incubation of the sample under dark conditions at ambient temperature, the absorbances were measured at 510 nm using a microplate reader. TFC was expressed as milligrams of rutin equivalents per gram of dry weight (mg RE/g DW).

### 2.5. Analysis of Major Secondary Metabolites

#### 2.5.1. Determination of Alkaloids and 3-Caffeoylquinic Acid

Alkaloids (caffeine and trigonelline) and 3-caffeoylquinic acid (3-CQA) were analyzed by high-performance liquid chromatography coupled with a diode array detector (HPLC-DAD) according to previously reported methods with minor modifications [8]. After ultrasonic extraction with aqueous methanol and centrifugation, the supernatant was subjected to chromatographic analysis. The separation process was accomplished by employing a reversed-phase C18 column, utilized in conjunction with a methanol-0.1% formic acid aqueous system under gradient elution conditions, maintained at a constant flow rate. The detection process was conducted at a wavelength of 254 nm for alkaloids and 325 nm for 3-CQA.

#### 2.5.2. Determination of Organic Acids

Organic acids were determined as described previously [8,11]. Briefly, ground coffee samples (2.50 g) were extracted with ultrapure water under reflux conditions, followed by centrifugation, dilution, and membrane filtration prior to analysis.

Succinic acid, citric acid, malic acid, acetic acid, lactic acid, tartaric acid, formic acid, and oxalic acid were quantified using HPLC-DAD. Chromatographic separation was performed on a Zorbax SB-AQ column using a methanol-phosphate buffer system under isocratic conditions. The detection process was conducted at a wavelength of 210 nm, and the quantification of the results was performed using external calibration with mixed standard solutions.

#### 2.5.3. Determination of Amino Acid

A quantity of 50.0 mg of the sample was meticulously combined with a solution of pre-cooled 70% methanol and water, as well as chloroform. The mixture was then subjected to a rigorous vortexing process, followed by a series of centrifugation steps, all of which were conducted at a low temperature. The resulting supernatant was subjected to further clarification by chilling and re-centrifugation, after which an aliquot was transferred to an autosampler vial and stored at −20 °C prior to analysis.

Quantification was performed using UPLC (ExionLC™ AD, Sciex, Framingham, MA, USA) coupled with tandem mass spectrometry (QTRAP^®^ 6500+, Sciex) equipped with an ACQUITY BEH Amide column. The separation process was accomplished through the utilization of a binary solvent system comprising aqueous ammonium acetate with formic acid and acetonitrile, incorporating the same additives, under gradient elution conditions. Electrospray ionization was operated in both positive and negative modes, and data were acquired in multiple-reaction-monitoring (MRM) mode. Amino acids were identified by comparison with an in-house library of authentic standards, and quantification was based on external calibration using MRM peak areas, ensuring high sensitivity and reproducibility.

### 2.6. Sensory Evaluation

The sensory evaluation of coffee was conducted by a panel of ten CQI-certified Q-Graders, following the Robusta specialty coffee cupping protocol established by the Uganda Coffee Development Authority (UCDA). As trained professional assessors performing routine cupping, the study was exempt from ethical review. All assessors provided informed consent. The coffee brew was prepared by steeping 8.50 g of ground coffee in 150.00 mL of hot water at 93 °C for 4 min. The sensory parameters evaluated included fragrance/aroma, flavor, salt/acidity, aftertaste, bitter/sweet, mouthfeel, balance, uniform cup, clean cup, and overall. The final score for each sample was the cumulative sum of the individual ratings across all taste characteristics.

### 2.7. UPLC-MS Analysis

Freeze-dried samples (50 mg) were subjected to a fine grinding process, followed by extraction with pre-cooled 70% methanol containing an internal standard. The extracts were subjected to intermittent vortexing, followed by centrifugation and clarification through membrane filtration. The supernatants were stored at −20 °C prior to analysis.

Non-volatile metabolites were analyzed using an ultra-high-performance liquid chromatography system (Vanquish, Thermo Scientific, Waltham, MA, USA) coupled with a Q Exactive HF-X mass spectrometer equipped with an electrospray ionization source. (Thermo Fisher Scientific, Waltham, MA, USA). The chromatographic separation was accomplished through the utilization of an ACQUITY HSS T3 column (Waters, Milford, MA, USA), employing water and acetonitrile as eluents. These liquids were acidified with formic acid to ensure optimal elution conditions. The acquisition of data was conducted in both positive and negative ionization modes, employing data-dependent MSn scanning.

Raw files were converted to mzML format and processed using XCMS for peak detection, alignment, and integration. Features with excessive missing values were removed, and the remaining gaps were imputed before normalization. Metabolite annotation was performed using an in-house database integrating public spectral libraries and met-DNA information. Only features meeting quality control criteria were retained, and results from both ionization modes were merged based on identification confidence.

The annotation of metabolites was conducted on the basis of accurate mass and MS/MS spectral comparison with in-house and public databases, corresponding to MSI level 2 identification (putatively annotated compounds). This MSI level 2 identification is widely accepted in the context of untargeted metabolomics.

### 2.8. Statistics and Analysis of Data

The assessment of intergroup differences was conducted through the implementation of a one-way analysis of variance (ANOVA) followed by a Duncan’s multiple range test, utilizing the SPSS Statistics 27.0 software (SPSS Inc., Chicago, IL, USA). Principal component analysis (PCA) and radar charts were generated in Origin 2021 (Origin Labs, Inc., Northampton MA, USA). Orthogonal partial least squares-discriminant analysis (OPLS-DA), permutation tests, weighted gene co-expression network analysis (WGCNA), multiple factor analysis (MFA), and clustering heatmaps were performed on the Metware Cloud data-processing platform (https://cloud.metware.cn, accessed on 12 April 2026). The measurement of each sample was conducted in triplicate, and the resulting data were reported as the mean ± standard deviation. Statistical significance was considered to have been achieved at *p* < 0.05.

## 3. Results and Discussion

### 3.1. The Effect of Different Primary Processing on the Taste Characteristics of Coffee Samples

**Figure 1:**
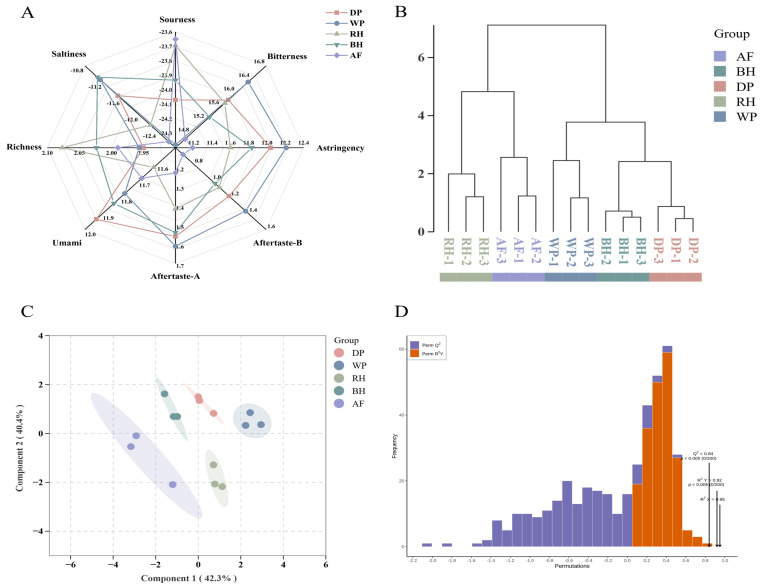
Analysis of electronic tongue taste characteristics. (**A**) Radar chart of electronic tongue taste characteristics. (**B**) Dendrogram of tree-based clustering using electronic tongue scores as the input matrix. (**C**) OPLS-DA analysis. (**D**) Permutation test model for OPLS-DA.

E-tongue is a device that utilizes chemical sensor arrays to generate objective, digital readouts with high sensitivity to taste stimuli [12]. Radar charts (Figure 1A) revealed distinct response profiles across processing methods. Notably, the sourness and saltiness levels for all samples were found to be below the reported human detection thresholds, aligning with prior observations [8]. The most significant disparities emerged in the dimensions of bitterness and astringency where the WP exhibited the most pronounced responses in both dimensions, while the anaerobic and honey-processed (red and black honey) samples demonstrated lower responses. The responses elicited by umami were found to be most intense in DP (11.94) and least intense in RH (11.62). This finding suggests that dry processing techniques may be more effective in preserving umami precursors.

To further resolve sample relationships, Hierarchical cluster analysis (HCA), OPLS-DA, and permutation tests were performed (Figure 1B–D). Multivariate analyses consistently revealed a clear separation among the five processing groups. The analysis of biological replicates revealed the presence of tight clustering, with between-group variation exhibiting a magnitude that was substantially greater in comparison to the variation present within each group. The OPLS-DA model exhibited a considerable proportion of explained variance and demonstrated success in passing permutation testing, thereby indicating adequate model robustness without manifest indications of overfitting.

### 3.2. Comparison of Total Polyphenol and Total Flavonoid Contents of Coffee Samples

As demonstrated in Table 1, the AF exhibited the highest TPC (60.24 mg GAE/g DW) and TFC (33.28 mg RE/g DW), which was in accordance with the hypothesis that anaerobic conditions impede phenolic oxidation, while microbial metabolism and acidic microenvironments facilitated the release of bound phenolics and flavonoids [4,13]. The DP was found to be the second most effective, which may be indicative of the hypothesis that prolonged contact between the pulp and the seed during the drying process facilitates the migration and retention of phenolics in the seed [14]. The results of this study indicated that both RH and BH exhibited intermediate levels, which may be attributed to prolonged exposure to high humidity during the process of honey production. It has been established that this condition is conducive to the degradation and oxidation of phenolics [15]. In contrast, the WP exhibited the lowest TPC (47.73 mg GAE/g DW) and TFC (23.50 mg RE/g DW), suggesting that the processes of washing and greater oxygen exposure during wet processing accelerate the loss and oxidation of soluble phenolics and flavonoids.

### 3.3. Analysis of Caffeine, 3-Caffeoylquinic Acid and Trigonelline in Coffee Samples

Caffeine, along with 3-CQA and trigonelline, are the most common bioactive in coffee and collectively shape taste. Caffeine accounts for 10–30% of perceived bitterness [16], while chlorogenic acid contributes to bitterness and acidity, particularly through decomposition and conversion into flavor-active compounds during roasting [17]. Trigonelline, an important flavor precursor, also participates in bitterness perception [18]. As summarized in Table 1, the levels of these three components varied among coffee beans produced by different primary processes. Caffeine ranged from 6.62 to 8.03 mg/g, with the highest level observed in AF (8.03 ± 0.08 mg/g) and the lowest in RH (6.62 ± 0.06 mg/g); DP (6.81 ± 0.04 mg/g) and WP (6.84 ± 0.12 mg/g) exhibited comparable caffeine contents, whereas BH contained 7.89 ± 0.11 mg/g. The 3-CQA content ranged from 8.90 to 11.66 mg/g, with the highest level in AF (11.66 ± 0.35 mg/g) and the lowest in WP (8.90 ± 0.35 mg/g). Trigonelline exhibited a narrower range (1.28–1.51 mg/g), being lowest in WP (1.28 ± 0.01 mg/g) and highest in BH (1.51 ± 0.05 mg/g). Overall, AF tended to exhibit higher levels of caffeine, 3-CQA, and trigonelline, whereas WP generally showed lower contents. However, the E-tongue results indicated that the WP exhibited the most pronounced bitterness and astringency, suggesting that other components contributed to its bitter properties.

### 3.4. Analysis of Organic Acids in Coffee Samples

Organic acids have been demonstrated to exert a substantial influence on the flavor profile of coffee. Previous research has established a robust correlation between coffee acidity and citric acid, malic acid, lactic acid, acetic acid, and related acids [19]. Consequently, the organic acid content of coffee beans with different primary processing was also quantified, and the result is shown in Table 2. The coffee beans that exhibited the highest total organic acid content (30.42 mg/g DW) in AF, whereas those processed by RH demonstrated the lowest (21.74 mg/g DW). These findings indicate that anaerobic processing increases organic acid levels in coffee, contributing to enhanced beverage quality and flavor.

Across the various treatments, citric, lactic, and formic acids were found to be the most prevalent, while oxalic acid was observed to be the least abundant. Citric acid was detected in all samples, with concentrations significantly higher in AF than in the other samples. The result indicates the potential for anaerobic fermentation conditions to facilitate citric acid accumulation. Higher citric acid levels in AF may enhance flavor diversity, as moderate citric acid contributes to the development of coffee’s acidity, berry notes, and fruity characteristics [20]. DP, WP, and RH exhibited consistent trends in citric, lactic, and malic acids, aligning with prior observations [21].

### 3.5. Analysis of Amino Acids in Coffee Samples

A total of 24 amino acids and related metabolites were identified in this study, as shown in Table 3. Overall, glutamic acid (Glu) and aspartic acid (Asp) exhibited the highest abundance in coffee bean samples, with concentrations ranging from 7.0 × 10^3^ to 1.8 × 10^4^ ng/g, significantly higher than other amino acids. Next were alanine (Ala), glycine (Gly), and tyrosine (Tyr), with concentrations generally at the 10^3^ ng/g level. Tryptophan (Trp), phenylalanine (Phe), valine (Val), and glutamine (Gln) exhibited moderate levels, while isoleucine (Ile), methionine (Met), and lysine (Lys) showed the lowest concentrations. Additionally, several non-protein amino acids and derivatives were detected, including γ-aminobutyric acid (GABA), β-alanine (β-Ala), and oxidized glutathione (GSSG), with GABA and glutathione exhibiting relatively high concentrations. Significant amino acid variations were observed across the five sample groups, with glutamic acid being the highest in DP, while aspartic acid and glycine levels were elevated in WP. Additionally, the higher concentrations of free amino acids (aspartic acid, glutamic acid, and alanine) in the WP were considered to be associated with protein hydrolysis, as they were required to provide raw materials for the germination process [22]. Conversely, the accumulation of γ-aminobutyric acid in DP may be related to responses to drought stress during prolonged exposure during drying [23].

In terms of taste perception, Glu and Asp are considered to impart umami and sourness, while Ala and Gly are associated with subtle sweetness. Aromatic amino acids like Phe and Tyr may contribute mild bitterness or astringency [24,25]. Compared to caffeine and chlorogenic acid, these amino acids are present in lower concentrations in coffee and thus have limited direct contribution to taste perception. However, these compounds serve as important precursors in Maillard and Strecker reactions during roasting. Variations in amino acid composition resulting from different primary processing methods may therefore influence the formation of key aroma-active compounds in roasted coffee. For instance, glycine, alanine, and branched-chain amino acids are associated with the generation of pyrazines and Strecker aldehydes, which contribute roasted, nutty, and malty notes. In contrast, sulfur-containing amino acids such as methionine and cysteine are involved in the formation of sulfur-containing volatiles that play a crucial role in coffee aroma [26,27].

### 3.6. Sensory Evaluation Analysis

As demonstrated in Figure S1, AF, RH and BH exhibited higher values compared to the traditional dry and wet treatments across all eight cup measure scores, consistent with previous findings [28]. Moreover, with the exception of flavor and mouthfeel, the BH obtained the highest scores for the remaining sensory indicators, demonstrating particularly noteworthy performance in the domains of balance, bitterness, sweetness, and overall quality. The underlying reason for this phenomenon is that anaerobic treatments indirectly contribute to the formation of aromatics through the process of microbial metabolism, which produces a variety of flavor precursors, including organic acids and ester precursors. The mellowness of the mouthfeel is attributed to an increase in metabolites such as glycerol and polysaccharides, among others. In addition, honey treatment has been shown to reduce roughness, enhance sweetness, and increase mellowness due to the retention of pectin and soluble saccharides [29]. Furthermore, uniform cup and clean cup were not reported in this instance due to the absence of variation across the different samples.

### 3.7. Metabolomic Analysis of Coffee Beans

#### 3.7.1. Overview on Coffee Bean Metabolites

**Figure 2:**
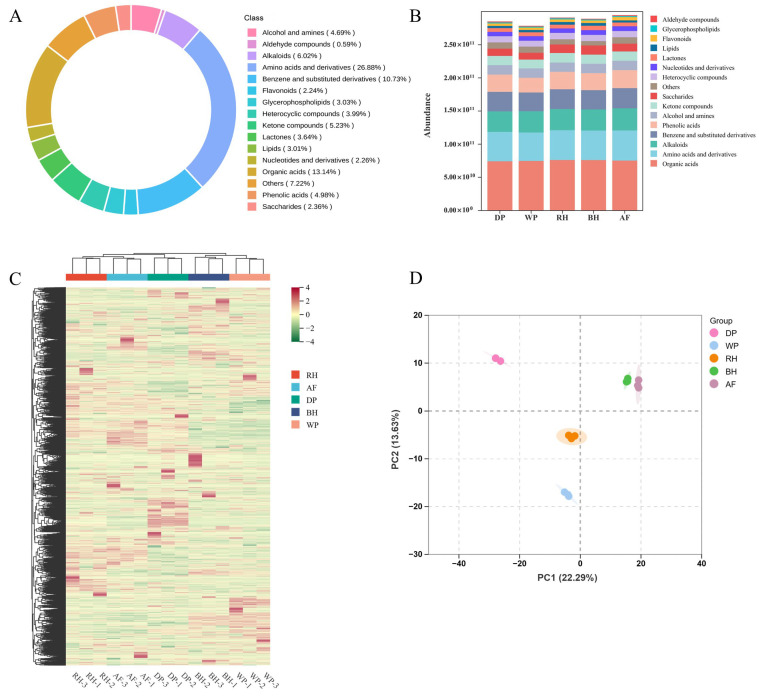
Metabolomic analysis of roasted coffee beans processed by different primary processing methods. (**A**) Metabolites composition. (**B**) Comparison of metabolite categories. (**C**) Hierarchical clustering analysis. (**D**) Distribution of samples from different primary processing treatments on a two-dimensional score plot of PCA.

A total of 5584 metabolites were identified across all coffee samples and categories with fewer than 100 metabolites were grouped as “other” in the summary statistics to enhance clarity, while original classifications were retained for subsequent differential metabolites (DMs) screening and MFA. Amino acids and derivatives were the most abundant, followed by organic acids and benzene derivatives (Figure 2A), underscoring their predominant role in the coffee metabolome.

As illustrated in Figure 2B, the stacked bar chart revealed that the AF exhibited the highest overall metabolite abundance. Specifically, the AF demonstrated approximately 1.24-fold and 1.19-fold higher phenolic acid and flavonoid contents, respectively, in comparison to the WP. This finding suggests that anaerobic conditions favor the retention of bioactive compounds, including phenolic acids and flavonoids [30,31]. The DP exhibited lower levels of organic acids, while the WP exhibited the lowest concentrations of amino acids, phenolic acids, and saccharides. Conversely, the presence of elevated organic acids and saccharides in RH and BH was observed, a phenomenon that can be attributed to the preservation of the mucilage layer during the honey processing. The preservation of mucilage layer provides fermentable substrates for microbial metabolism, thereby influencing the observed differences in the composition of these samples. These substrates can undergo a transformation into organic acids during the fermentation process and further modified via enzymatic or secondary reactions during the drying stage, thereby promoting accumulation and retention [21,28]. Collectively, these findings indicate that primary processing can significantly influence the levels of phenolic acids, flavonoids, amino acids, sugars, and organic acids in coffee beans, thereby imparting corresponding flavors to the coffee.

As illustrated in Figure 2C, HCA revealed significant disparities in metabolite abundance, manifesting as distinct up- and down-regulated patterns and tight clustering of biological replicates. This observation signifies a high degree of data reliability. In the two-dimensional PCA score plot (Figure 2D), DP was distinctly separated along PC1 (22.29%), WP shifted primarily along PC2 (13.36%), while AF, DH, and RH were positioned on the positive side of PC1. Overall, AF and DH demonstrated a higher degree of similarity, while DP exhibited the most significant divergence, underscoring pronounced intergroup disparities in metabolic profiles.

#### 3.7.2. Analysis of Differential Metabolites Identified in Coffee Beans

Multivariate OPLS-DA, a supervised method that provides clearer visual separation in score space than PCA, was applied to the dataset. The OPLS-DA score plot (Figure S2A) showed distinct separation of the five processing groups, with compact clustering of biological replicates. The associated 200-run permutation test (Figure S2B) indicated a robust model (R^2^Y = 0.998, Q^2^ = 0.825, *p* < 0.005). Using VIP > 1, *p* < 0.05, and FDR < 0.05 as selection criteria, 808 DMs were identified. Subsequent hierarchical clustering (Figure S2C) grouped the samples by processing method, confirming the presence of clear intergroup differences in metabolic profiles.

To further investigate distinctions among different primary processing treatments, DP was fixed as the control group and pairwise comparisons were conducted with the remaining four sample groups. Pairwise comparisons between DP and the other processing treatments revealed clear metabolic divergence (Figures S3 and S4). Across all contrasts involving DP, down-regulated metabolites consistently outnumbered up-regulated ones. The DP-BH and DP-AF comparisons demonstrated the most significant overall discrepancies, while DP-RH exhibited the least variation, consistent with their PCA proximity. Although specific metabolite counts and identities varied, lipid-related metabolites-including fatty acids and phospholipid-and amino acid derivatives accounted for a substantial proportion of the DMs. These compound-level trends are consistent with the processing-induced chemical transformations described in the literature, including lipid oxidation, phospholipid remodeling, and amino acid-derived flavor precursor formation (see Supplementary Materials Figures S3 and S4 for additional analysis).

#### 3.7.3. KEGG Pathway Enrichment Analysis

**Figure 3:**
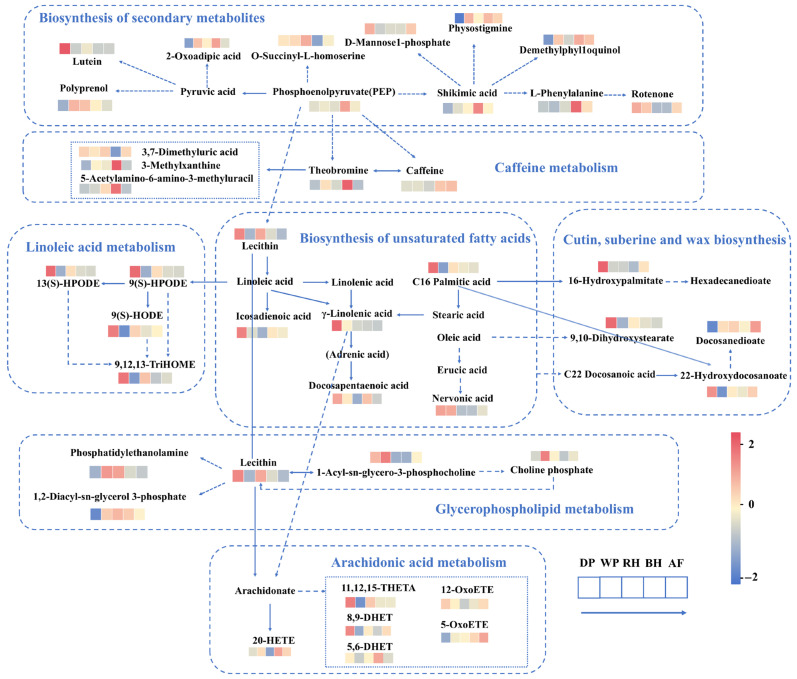
Summary of significantly enriched KEGG pathways across different coffee processing methods. Pathways with *p* < 0.05 and matched metabolites ≥3 from the top 20 KEGG pathways in each pairwise comparison were included. Solid arrows indicate direct, one-step reactions, whereas dashed arrows indicate indirect formation via multistep reactions.

Coffee beans subjected to different primary processing methods exhibited significant variations in their metabolic chemical fingerprints. Although roasting temperatures inactivate biological enzymes, the pathway enrichment of residual compounds in the roasted product enables retrospective mapping to the original metabolic networks. Specifically, the high residual abundance of certain metabolites in the roasted coffee beans indicates either their accumulation during the green bean stage or their differential degradation during roasting, thereby serving as a metabolic footprint of the primary processing (Figure 3). From the top 20 KEGG pathways in each processing comparison, only those with *p* < 0.05 and matched metabolites ≥3 were considered significantly enriched.

Two lipid-related KEGG pathways, biosynthesis of unsaturated fatty acids and cutin, suberin, and wax biosynthesis, were significantly enriched in all four pairwise comparisons. The former aids in stabilizing membrane structures, helping plants adapt to environmental stress, while the latter is associated with constructing protective epidermal barriers [32,33]. Notably, compared to samples treated with other methods, most metabolites in these two pathways were upregulated in DP sample. One possible explanation is that the DP sample was exposed to sunlight for extended periods, enduring more intense environmental stress, which prompted the plant cells to adopt self-protective measures. These measures include increased synthesis of unsaturated fatty acids to stabilize membrane structures, accumulation of cutin and wax components to reinforce the epidermal barrier, and activation of stress signaling pathways to promote the synthesis of these metabolites [32].

In most pairwise comparisons, linoleic acid metabolism and arachidonic acid metabolism lipid oxidation-related were significantly enriched. Green coffee beans possess high lipid content and are rich in unsaturated fatty acids. Prolonged drying during processing continuously exposes the beans to oxygen and heat, triggering the oxidation of unsaturated fatty acids. From a metabolomic perspective, this transformation manifests as significant enrichment in the linoleic acid and arachidonic acid metabolic pathways. Linoleic acid oxidation yields hydrogen peroxides such as 13(S)-HPODE and 9(S)-HPODE, along with their reduced products 9(S)-HODE and 9,12,13-TriHOME; Arachidonic acid, metabolized via the cytochrome P450 pathway, yields oxidation products including 20-HETE, 8,9-DHET, and 5-OxoETE. The accumulation of these metabolites reflects the extensive impact of processing on the oxidative state of coffee bean fatty acids.

Although common trends in lipid changes exist across processing methods, the comparison between WP and DP is the only scenario showing significant enrichment of the glycerophospholipid metabolic pathway, indicating that wet processing induces pronounced alterations in membrane lipids [34]. In the wet process, coffee beans are fermented in water and thoroughly rinsed after hulling. This process may degrade membrane phospholipids through three mechanisms: (1) Natural enzymes and microbial phospholipases hydrolyze membrane phospholipids, releasing fatty acids. Previous studies have reported that yeasts such as *Saccharomyces cerevisiae* and *Pichia kudriavzevii* are involved in coffee fermentation and may contribute to mucilage degradation and the formation of flavor-related metabolites during processing [11,29]. (2) The warm, humid environment may trigger transient germination-like metabolism in coffee beans, consuming membrane lipids and activating lipases. During seed germination, phospholipase activity significantly increases, leading to degradation of membrane phospholipid molecules and restructuring of membrane structure. (3) Subsequent washing steps further elute water-soluble degradation products. Similar phenomena have been observed in cocoa bean fermentation studies. During fermentation, postmortem changes occur, including cell membrane disruption, followed by the release of storage components from within the cell [35].

In contrast, sun-dried coffee beans ferment within intact fruit, which potentially shields cellular structures from direct microbial assault and rapid hydration. Initial dry conditions also prevent the triggering of germination and membrane renewal processes.

Therefore, the unique enrichment of glycerophospholipid metabolic pathways in WP can be attributed to two mechanisms: the action of microbial enzymes and the metabolic reactions of the coffee beans themselves during wet fermentation. These reactions disrupt cell membranes and lead to the selective loss or alteration of phospholipids. This may impact the final flavor and quality because phospholipids and their degradation products influence the Maillard reaction and the formation of aroma precursors during roasting. The interaction between the Maillard reaction and lipid oxidation generates heterocyclic compounds, such as furans, pyrazines, and thiophenes, which significantly enhance coffee aroma complexity [36].

The pathways of secondary metabolite biosynthesis and caffeine metabolism were significantly enriched in the comparison of the BH and DP. This indicates that the BH group exhibited substantial alterations in multiple phytochemicals, including alkaloids (e.g., caffeine and theobromine), terpenoids (e.g., polyvinyl alcohol), and phenolic precursors (e.g., shikimic acid and phenylalanine), compared to the DP. This shift reflects the fermentation characteristic of BH, which is supported by a substantial mucilage layer that sustains microbial activity (e.g., yeast, lactic acid bacteria, and acetic acid bacteria) for a much longer period than rapid drying processes. During fermentation, yeast produces volatile metabolites such as higher alcohols, esters, and aldehydes, while bacteria primarily generate organic acids (e.g., lactic acid and acetic acid). These metabolites permeate the interior of the coffee bean and serve as flavor precursors that transform into sweet, fruity, and caramel-like aromas during roasting [20,28]. Furthermore, the significant enrichment of caffeine metabolism indicates that BH processing creates a unique biochemical environment that promotes the accumulation of caffeine and its metabolites unlike DP. The mucilage layer retained in BH provides a rich carbon source for abundant microorganisms. These microorganisms convert caffeine into theobromine, 3-methylxanthine, 3,7-dimethyluric acid, and other intermediates via N-demethylation and partial oxidation. Additionally, upstream phosphoenolpyruvate may indirectly convert into caffeine and theobromine.

Overall, variations in microbial communities and enzyme activities induced by distinct processing conditions appear to be major drivers of these pathway-specific metabolic shifts. Primary processing methods thus constitute a dominant factor shaping the chemical composition of coffee beans, mediating flavor development through diverse microbial and biochemical routes [21]. In particular, the enrichment of lipid- and phospholipid-related pathways suggests that different primary processing methods may alter the availability of lipid-derived precursors and membrane-related substrates, which can further interact with Maillard and other thermal reactions during roasting. Therefore, although volatile compounds were not directly analyzed in this study, the observed processing-induced metabolic shifts may still be relevant to the potential formation of roast-related flavor compounds.

### 3.8. Association of Metabolite Profiles with Important Sensory Properties by WGCNA

**Figure 4:**
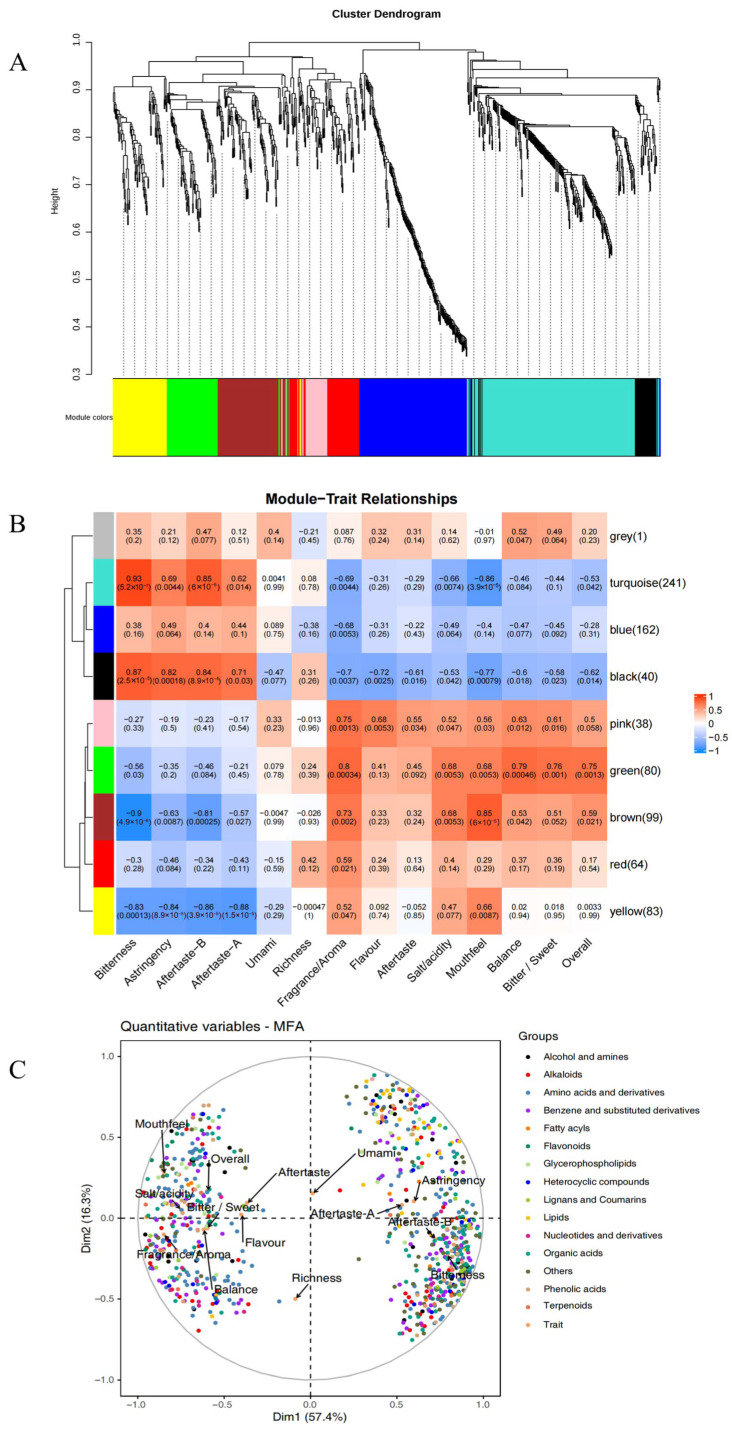
Correlation analysis of differential coffee metabolites with key sensory traits based on WGCNA. (**A**) Average network adjacency clustering branch diagram for identifying metabolite co-expression modules. (**B**) Module-trait correlation heatmap. (**C**) Multifactor analysis (MFA). Colored dots represent different metabolite classes, and the trait variables are additionally indicated by arrows and labels in the plot.

We employed WGCNA to construct a scale-free topological network, thereby investigating the relationships among 808 DMs and taste characteristics as determined by E-tongue and sensory evaluation measurements. E-tongue measurements (bitterness, astringency, aftertaste-B, aftertaste-A, umami, richness) and sensory evaluations (fragrance/aroma, flavor, salt/acidity, aftertaste, bitter/sweet, mouthfeel, balance, and overall) were utilized. As shown in Figure 4A, each vertical branch represents a metabolite, with colored bars at the base indicating module partitions obtained via dynamic tree cut. Eight modules were identified (turquoise, blue, black, pink, green, brown, red, and yellow) and modules with |r| > 0.6 and *p* < 0.05 were identified as flavor-associated in this study. The module-trait heatmap (Figure 4B) demonstrated that the turquoise and black modules exhibited positive correlations with bitterness and aftertaste-B (r > 0.8, *p* < 0.001) and negative correlations with mouthfeel (r < −0.75, *p* < 0.001). Conversely, bitterness and astringency were significantly negatively correlated with brown and yellow modules (r < −0.8, *p* < 0.001). Additionally, the brown module displayed a substantial positive correlation with mouthfeel (r > 0.8, *p* < 0.001). Pink and green modules significantly connected with mouthfeel (r > 0.7, *p* < 0.01), whereas the green module also positively correlated with balance, bitter/sweet, and overall (r > 0.7, *p* < 0.01).

To further validate the reliability of WGCNA, MFA was performed on 744 metabolites from key modules (turquoise, blue, black, pink, green, brown, yellow) with |r| > 0.6 and *p* < 0.05 (Figure 4C). The loading plot illustrated that the first two main components of MFA explained 73.7% of the variance (Dim1 = 57.4%, Dim2 = 16.3%), with 86.1% of metabolites obtaining absolute values > 0.5 on Dim1. These metabolites were distributed across both ends of the horizontal axis and showed strong correlations with key taste characteristics including bitterness, astringency, aftertaste-B, aftertaste-A, umami, richness, fragrance/aroma, and mouthfeel. MFA results validated the reliability of WGCNA.

Building upon WGCNA, we further screened key metabolites within candidate modules (|r| > 0.6, *p* < 0.05) using dual thresholds gene significance (GS) > 0.6 and (module membership) |MM| > 0.8 to identify core components potentially driving module-trait relationships [37]. A total of 467 metabolites were screened, mainly including 107 amino acids and derivatives, 62 organic acids, 60 Benzene and substituted derivatives, 30 alkaloids and 27 phenolic acids. Hierarchical cluster heatmaps were generated for amino acids and derivatives, organic acids, Benzene and substituted derivatives, alkaloids, and phenolic acids to visualize their content variations across five groups of differently processed samples (see Supplementary Materials Figure S5A–E for additional analysis).

### 3.9. Analysis of Key Metabolites Associated with Taste Characteristics of Coffee Under Different Primary Processing Methods

**Figure 5:**
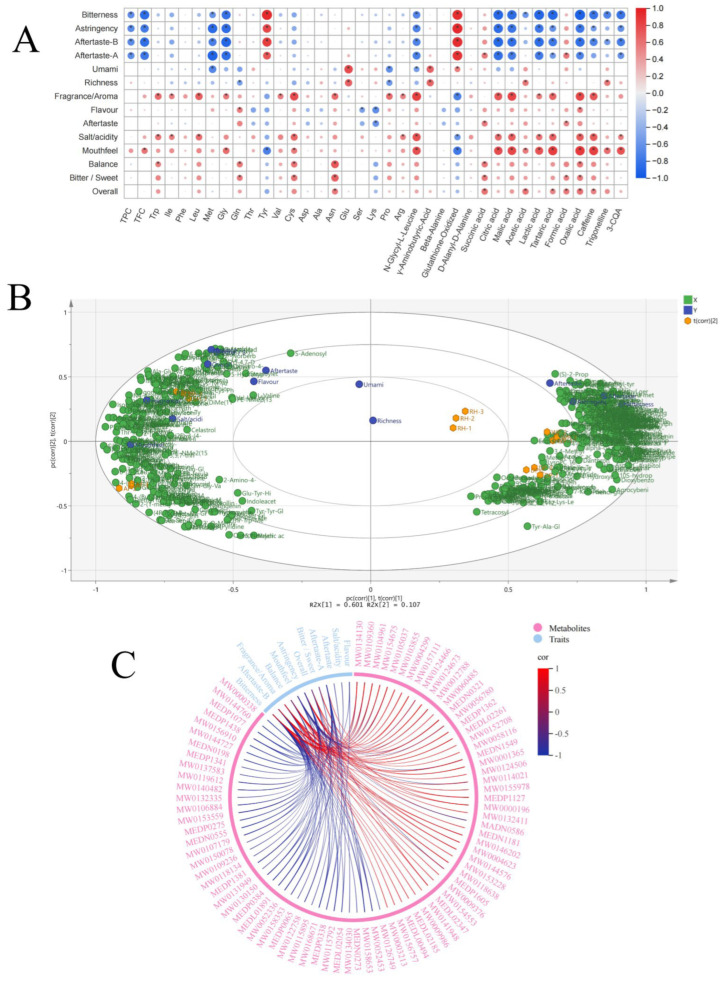
Taste-metabolite association in the five kinds of coffee beans under different primary processing methods. (**A**) Heatmap representing the pearson correlation analysis between their taste scores and main active compounds/secondary metabolites. * indicates statistical significance at *p* < 0.05. Larger dots indicate stronger correlations, while color intensity reflects the strength and direction of the correlation. (**B**) Score plot of the orthogonal partial least squares discriminant analysis; R^2^X [1] = 60.1%, R^2^X [2] = 10.7%. (**C**) Chord plot representing the Pearson correlation analysis between taste scores and metabolites.

Coffee quality is conventionally evaluated from physical, chemical, and sensory perspectives. Physical assessment focuses on green-bean characteristics such as size, shape, and defect incidence, whereas chemical assessment identifies and quantifies key nonvolatile compounds, including caffeine, 3-CQA, trigonelline, organic acids and amino acids. During roasting, these precursors undergo Maillard and related thermal reactions, giving rise to volatile flavor compounds that define coffee aroma. At the same time, they directly contribute to taste and aroma perception, collectively shaping the overall flavor profile of coffee [38]. To investigate the relationship between key taste characteristics of coffee and major secondary metabolites, Pearson correlation analysis was employed to determine the association between 14 important sensory characteristics and 37 compounds (Figure 5A). Red and blue represent positive and negative correlations, respectively, with darker shades indicating stronger correlations. Figure 5A indicates that taste characteristics such as bitterness, aftertaste-B, and mouthfeel in coffee are closely correlated with caffeine, 3-CQA, and trigonelline content. Citric acid, malic acid, lactic acid, tartaric acid, and oxalic acid showed significant negative correlations with bitterness, astringency, and aftertaste-A/B, while positively correlating with salt/acidity, suggesting organic acids partially mitigate bitterness and balance acidity through mixture suppression (Breslin, 1996) [39]. Glutathione-oxidized and Tyr were found to be significantly positively correlated with bitterness, astringency, and their aftertaste. TPC, TFC, Gly, and Met were significantly negatively correlated with bitterness, astringency, and their aftertaste. Glu exhibited a positive correlation with umami, consistent with previous research studies [24].

Partial Least Squares Regression (PLSR) is a multivariate regression method commonly used to analyze data with multiple independent and dependent variables. PLSR is particularly suitable when the number of independent variables far exceeds the sample size, can handle multiple response variables, and avoids overfitting by reducing feature dimensions [40].

To investigate the influence of metabolomic profiles on key taste characteristics in coffee samples, a PLSR model was established using the relative abundance of 467 untargeted metabolites as independent variable X and 14 key taste characteristics as dependent variable Y (Figure 5B). The biplot visually reflects the distribution patterns of different sample groups in latent variable space and their relationships with each sensory attribute. Figure 5B shows that PC1 serves as the primary discriminating axis (R^2^X [1] = 60.1%, R^2^X [2] = 10.7%). Variables aligned with the vector and closer to the outer circle exhibit stronger correlations with this principal component, while opposite directions indicate negative correlations. Results indicate that WP and DP predominantly project onto the positive direction of PC1, aligning with bitterness, astringency, Aftertaste-A, and Aftertaste-B. BH and AF primarily reside on the negative direction of PC1, aligning with mouthfeel, fragrance/aroma, flavor, bitter/sweet, and salt/acidity. RH approach the origin, exhibiting a relatively balanced sensory phenotype. This result aligns with E-tongue and sensory evaluation findings, suggesting a consistent association between chemical variation and taste-related characteristics.

At the chemical level, the positive PC1 dimension primarily enriched small-molecule peptides and aromatic acids, correlating with stronger bitterness and astringency; the negative PC1 dimension enriched flavonoids, alkaloids, and certain organic acids, aligning with enhanced aroma and mouthfeel. The DP dimension was associated with variations in short-chain acids, aldehydes, polyols, and sugar phosphates. RH, being near the origin, did not exhibit a clearly dominant component. Eighty-two key taste compounds were identified using three criteria (VIP > 1, Pearson |r| > 0.8, *p* < 0.001) and analyzed via Pearson correlation chord plot (Figure 5C). Results indicate that coffee bitterness shows significant positive correlations with 1,3,4,5-tetrahydroxycyclohexanecarboxylic acid, tyrosol, and prolyl-glutamine, while exhibiting significant negative correlations with Ala-Trp-Glu, Glu-Ala-Trp, and citric acid. Aftertaste-B was significantly positively correlated with 5,7-dihydroxy-3-(2-hydroxy-3,4-dimethoxyphenyl)-3,4-dihydro-2H-1-benzopyran-4-one, 3-amino-2,3-dihydrobenzoic acid, and prolyl-glutamine, while showing significant negative correlations with diethyl fumarate, 2-(1-methyl-1H-imidazol-4-yl)acetic acid, and Tyr-Tyr-Ser. Astringency showed significant positive correlations with 3-hydroxydecanoic acid, (E)-ligustilide, and 2-methyl citric acid, while exhibiting significant negative correlations with carnitine C6:1, Met-Gly-Phe, and Ala-Ser-Gln. Aftertaste-A showed a significant positive correlation with 1,3,4,5-tetrahydroxycyclohexanecarboxylic acid and a significant negative correlation with 2′-O-methyladenosine, Ala-Ser-Gln, hydroxyphenyllactic acid, etc. Fragrance/aroma showed a significant positive correlation with 3-hydroxycinnamic acid, 10S-hydroperoxy-8E-octadecenoic acid, and methyl behenate, while showing significant negative correlations with 4,4′-oxydianiline, 1,4-dibenzylpiperazine, and 4-amino-5-(4-methoxybenzyl)-4H-1,2,4-triazole-3-thiol. Balance showed significant positive correlations with Ala-Glu-Val-Asp and L-phenylalanine, and significant negative correlations with Tyr-Ala-Glu-Leu-Arg and Glu-Lys-Leu-Thr-His. Overall showed significant positive correlations with sodium salicylate and 1-acetylindole, and significant negative correlations with agrocybenine and (R)-quinuclidin-3-amine.

## 4. Conclusions

Coffee samples were comprehensively characterized by combining E-tongue analysis with UPLC-MS to elucidate the effects of primary processing on metabolomics and taste characteristics, while revealing relationships between chemical composition and flavor quality. In E-tongue analysis, DP exhibited the strongest response to the umami sensor, while WP demonstrated the highest bitterness, astringency, and aftertaste among all coffee samples. Major secondary metabolites in coffee beans were quantified, revealing that different primary processing methods significantly influenced the content of caffeine, 3-CQA, organic acids, and most amino acids, indicating distinct quality profiles among processed beans.

Multigroup metabolomic analysis identified 808 DMs across the five treatments. WGCNA further identified eight co-expression modules and 467 hub metabolites, which were primarily amino acids and derivatives, organic acids, alkaloids, and phenolic acids. Using DP as the reference, KEGG enrichment of pairwise contrasts with WP, RH, BH and AF highlighted lipid remodeling. Key enriched pathways included those related to lipid metabolism (e.g., biosynthesis of unsaturated fatty acids; cutin, suberin, and wax biosynthesis) across all treatments. Notably, glycerophospholipid metabolism was specifically enriched in WP compared to DP, whereas caffeine metabolism and other secondary metabolite biosynthesis pathways were predominantly associated with BH compared to DP. Correlation analysis and PLSR further linked several metabolites (such as 1,3,4,5-tetrahydroxycyclohexanecarboxylic acid, citric acid, tyrosine, TPC and TFC, and glutamic acid) to bitterness, mouthfeel, umami, and aftertaste traits. These findings provide insights into the regulatory mechanisms of processing methods on quality formation, thereby offering scientific support for optimizing primary processing techniques and enhancing product sensory quality.

Nevertheless, this study was based on coffee samples from a single origin and primarily focused on non-volatile metabolites. Future studies incorporating samples from diverse origins, as well as integrating volatile compound analysis, would further enhance the understanding of coffee flavor formation.

## Figures and Tables

**Table 1:** Variation in active compounds among coffee beans processed by different primary processing methods.

No	Content (mg/g)	DP	WP	RH	BH	AF
1	TPC	57.89 ± 2.13 ^ab^	47.73 ± 3.54 ^d^	54.27 ± 1.462 ^bc^	51.71 ± 2.82 ^cd^	60.24 ± 1.64 ^a^
2	TFC	27.23 ± 1.49 ^b^	23.50 ± 2.09 ^c^	25.88 ± 0.96 ^bc^	26.37 ± 1.96 ^b^	33.82 ± 0.91 ^a^
3	Caffeine	6.81 ± 0.04 ^b^	6.84 ± 0.12 ^b^	6.62 ± 0.06 ^c^	7.89 ± 0.11 ^a^	8.03 ± 0.08 ^a^
4	3-CQA	10.29 ± 0.57 ^b^	8.90 ± 0.35 ^c^	9.13 ± 0.60 ^c^	10.25 ± 0.26 ^b^	11.66 ± 0.35 ^a^
5	Trigonelline	1.48 ± 0.01 ^a^	1.28 ± 0.01 ^c^	1.34 ± 0.04 ^b^	1.51 ± 0.05 ^a^	1.49 ± 0.01 ^a^

Samples labeled with different superscript letters in the same column differ significantly from one another (*p* < 0.05).

**Table 2:** Variation in organic acids among coffee beans processed by different primary processing methods.

No	Content (mg/g)	DP	WP	RH	BH	AF
1	Succinic acid	0.34 ± 0.01 ^bc^	0.32 ± 0.02 ^c^	0.35 ± 0.019 ^b^	0.37 ± 0.02 ^a^	0.29 ± 0.01 ^d^
2	Citric acid	10.86 ± 0.59 ^d^	11.62 ± 0.55 ^cd^	12.43 ± 0.38 ^c^	13.85 ± 0.64 ^b^	18.08 ± 0.58 ^a^
3	Malic acid	0.83 ± 0.05 ^d^	0.90 ± 0.04 ^c^	0.97 ± 0.03 ^c^	1.11 ± 0.05 ^b^	1.28 ± 0.04 ^a^
4	Acetic acid	0.64 ± 0.02 ^a^	0.46 ± 0.02 ^d^	0.53 ± 0.03 ^c^	0.65 ± 0.03 ^a^	0.60 ± 0.02 ^b^
5	Lactic acid	5.47 ± 0.26 ^bc^	5.15 ± 0.28 ^c^	5.35 ± 0.16 ^bc^	5.69 ± 0.26 ^b^	6.52 ± 0.21 ^a^
6	Tartaric acid	0.19 ± 0.01 ^c^	0.16 ± 0.01 ^d^	0.17 ± 0.01 ^d^	0.26 ± 0.01 ^b^	0.29 ± 0.01 ^a^
7	Formic acid	3.72 ± 0.31 ^ab^	3.41 ± 0.16 ^bc^	3.70 ± 0.20 ^ab^	3.98 ± 0.18 ^a^	3.12 ± 0.10 ^c^
8	Oxalic acid	0.150 ± 0.00 ^c^	0.134 ± 0.01 ^cd^	0.174 ± 0.01 ^d^	0.228 ± 0.01 ^b^	0.254 ± 0.01 ^a^

Samples labeled with different superscript letters in the same column differ significantly from one another (*p* < 0.05).

**Table 3:** Variation in amino acids and derivatives among coffee beans processed by different primary processing methods.

No	Content (ng/g)	DP	WP	RH	BH	AF
1	Trp	588.20 ± 34.48 ^c^	679.49 ± 14.78 ^b^	633.30 ± 31.70 ^c^	747.01 ± 22.74 ^a^	700.91 ± 22.47 ^b^
2	Ile	18.14 ± 2.58 ^d^	52.82 ± 5.51 ^b^	20.45 ± 2.89 ^d^	43.22 ± 4.29 ^c^	75.01 ± 5.52 ^a^
3	Phe	162.70 ± 5.07 ^b^	212.77 ± 4.27 ^a^	178.61 ± 6.01 ^b^	204.98 ± 18.62 ^a^	202.13 ± 9.88 ^a^
4	Leu	104.12 ± 10.80 ^b^	155.48 ± 10.69 ^a^	119.76 ± 11.63 ^b^	168.55 ± 10.87 ^a^	161.91 ± 10.34 ^a^
5	Met	14.98 ± 2.10 ^c^	16.32 ± 0.64 ^c^	36.81 ± 1.88 ^b^	13.86 ± 1.41 ^c^	51.78 ± 4.88 ^a^
6	Gly	926.80 ± 50.27 ^c^	1015.26 ± 22.35 ^bc^	1050.33 ± 56.11 ^b^	1085.44 ± 24.79 ^b^	1444.51 ± 106.43 ^a^
7	Gln	300.20 ± 12.91 ^c^	426.84 ± 26.67 ^ab^	510.50 ± 28.32 ^a^	461.12 ± 31.81 ^a^	344.47 ± 16.97 ^bc^
8	Thr	49.71 ± 6.48 ^c^	125.67 ± 8.69 ^a^	9.30 ± 1.18 ^d^	99.80 ± 13.90 ^b^	134.02 ± 14.47 ^a^
9	Tyr	953.37 ± 43.74 ^bc^	1539.05 ± 123.29 ^a^	989.79 ± 28.85 ^b^	848.01 ± 45.84 ^c^	696.51 ± 17.29 ^d^
10	Val	200.54 ± 19.47 ^b^	344.15 ± 19.12 ^a^	217.78 ± 19.26 ^b^	360.98 ± 33.73 ^a^	349.38 ± 26.49 ^a^
11	Cys	509.20 ± 8.49 ^d^	565.13 ± 19.85 ^bc^	557.06 ± 7.75 ^c^	599.25 ± 9.33 ^a^	588.70 ± 25.57 ^ab^
12	Asp	6859.34 ± 66.08 ^e^	9698.50 ± 158.15 ^a^	7224.74 ± 105.22 ^d^	8207.56 ± 145.55 ^c^	9130.46 ± 186.47 ^b^
13	Ala	2235.15 ± 137.72 ^ab^	2331.53 ± 173.12 ^a^	2029.58 ± 176.67 ^b^	2425.37 ± 119.90 ^a^	2376.07 ± 28.62 ^a^
14	Asn	302.83 ± 15.51 ^c^	661.84 ± 19.51 ^b^	618.71 ± 34.99 ^b^	1093.96 ± 113.26 ^a^	566.81 ± 63.75 ^b^
15	Glu	12,439.1 ± 570.38 ^cd^	18,414.58 ± 815.20 ^a^	11,737.31 ± 1068.89 ^d^	14,917.28 ± 938.33 ^b^	13,487.55 ± 377.14 ^c^
16	Ser	327.87 ± 13.85 ^c^	561.85 ± 13.28 ^a^	179.98 ± 21.07 ^d^	467.65 ± 13.22 ^b^	558.58 ± 20.02 ^a^
17	Lys	115.84 ± 8.60 ^b^	105.45 ± 10.98 ^b^	18.91 ± 1.48 ^d^	49.92 ± 12.16 ^c^	194.00 ± 13.74 ^a^
18	Pro	NA	959.38 ± 31.48 ^a^	684.08 ± 14.79 ^c^	829.00 ± 47.51 ^b^	809.76 ± 24.97 ^b^
19	Arg	NA	148.09 ± 12.21 ^b^	NA	180.58 ± 13.29 ^a^	116.90 ± 13.79 ^c^
20	N-Glycyl-L-Leucine	477.44 ± 19.78 ^c^	534.39 ± 13.08 ^b^	541.46 ± 33.93 ^b^	564.12 ± 22.02 ^b^	628.46 ± 17.47 ^a^
21	γ-Aminobutyric-Acid	21,515.56 ± 529.11 ^a^	4218.25 ± 149.59 ^d^	5995.99 ± 34.44 ^c^	11,137.26 ± 885.58 ^b^	6353.70 ± 105.83 ^c^
22	Beta-Alanine	1135.60 ± 34.34 ^a^	1207.58 ± 57.90 ^a^	1117.68 ± 51.53 ^a^	1154.29 ± 64.35 ^a^	1173.42 ± 71.61 ^a^
23	Glutathione-Oxidized	27,241.45 ± 631.05 ^a^	26,369.92 ± 508.53 ^b^	18,962.55 ± 361.23 ^c^	18,126.09 ± 167.57 ^d^	12,445.35 ± 622.49 ^e^
24	D-Alanyl-D-Alanine	35.02 ± 2.51 ^b^	41.27 ± 3.53 ^a^	33.74 ± 2.79 ^b^	38.34 ± 2.26 ^ab^	38.25 ± 3.55 ^ab^

Samples labeled with different superscript letters in the same column differ significantly from one another (*p* < 0.05). NA indicates that the analyte was not detected in the sample because its concentration was below the limit of detection (LOD).

## Data Availability

The original contributions presented in this study are included in the article/Supplementary Materials. Further inquiries can be directed to the corresponding authors.

## Supplementary Materials

The following supporting information can be downloaded at: https://www.mdpi.com/article/10.3390/foods15091475/s1, Figure S1. Radar graph of coffee samples sensory attributes; Figure S2. Multivariate analysis and hierarchical clustering of coffee samples based on primary processing methods. (A) OPLS-DA score plot showing distinct separation of the five groups with tight clustering of biological replicates. (B) Permutation test (200 permutations) confirming model robustness (R^2^Y = 0.998, Q^2^ = 0.825, *p* < 0.005). (C) Heatmap from hierarchical clustering of differential metabolites, highlighting clear metabolic differences between processing methods; Figure S3. Validation and visualization of differential metabolites. (A) Model validation using the DP group as the reference. (B) Permutation test (200 runs) to confirm model robustness. (C) Volcano plot of differential metabolites (FC ≥ 2 or ≤0.5, VIP > 1, *p* < 0.05). (D) Bar chart of the top 20 differential metabolites based on fold change. (E) Pathway enrichment analysis of differential metabolites (The order of paired comparisons from top to bottom is: WP vs. DP, RH vs. DP, BH vs. DP, AF vs. DP); Figure S4. Venn diagram of differential metabolites among pairwise comparisons; Figure S5. Heatmap using key differential metabolites identified by targeted metabolomic and WGCNA analyses as the input matrix. (A) Amino acids and derivatives (*n* =107). (B) Organic acids (*n* = 62). (C) Benzene and its derivatives (*n* = 60). (D) Alkaloids (*n* = 30). (E) Phenolic acids (*n* = 27).

## Abbreviations

WGCNA	weighted gene co-expression network analysis
UPLC-MS	ultra-performance liquid chromatography-mass spectrometry
QC	quality control samples
*p*	probability value
VIP	variable importance in the projection
HPLC-DAD	high-performance liquid chromatography-diode array detector
OPLS-DA	orthogonal partial least squares discriminant analysis
FDR	false discovery rate
FC	fold change
r	correlation coefficient

## References

[1] Freitas V.V., Borges L.L.R., Vidigal M.C.T.R., dos Santos M.H., Stringheta P.C. (2024). Coffee: A Comprehensive Overview of Origin, Market, and the Quality Process. Trends Food Sci. Technol..

[2] Barreto Peixoto J.A., Silva J.F., Oliveira M.B.P.P., Alves R.C. (2023). Sustainability Issues along the Coffee Chain: From the Field to the Cup. Compr. Rev. Food Sci. Food Saf..

[3] Chan M.Z.A., Lau H., Lim S.Y., Li S.F.Y., Liu S.-Q. (2021). Untargeted LC-QTOF-MS/MS Based Metabolomics Approach for Revealing Bioactive Components in Probiotic Fermented Coffee Brews. Food Res. Int..

[4] Várady M., Tauchen J., Klouček P., Popelka P. (2022). Effects of Total Dissolved Solids, Extraction Yield, Grinding, and Method of Preparation on Antioxidant Activity in Fermented Specialty Coffee. Fermentation.

[5] Febrianto N.A., Zhu F. (2023). Coffee Bean Processing: Emerging Methods and Their Effects on Chemical, Biological and Sensory Properties. Food Chem..

[6] Zhang W., Bai B., Du H., Hao Q., Zhang L., Chen Z., Mao J., Zhu C., Yan M., Qin H. (2024). Co-Expression of Metabolites and Sensory Attributes through Weighted Correlation Network Analysis to Explore Flavor-Contributing Factors in Various *Pyrus* Spp. Cultivars. Food Chem. X.

[7] Fan F.-Y., Huang C.-S., Tong Y.-L., Guo H.-W., Zhou S.-J., Ye J.-H., Gong S.-Y. (2021). Widely Targeted Metabolomics Analysis of White Peony Teas with Different Storage Time and Association with Sensory Attributes. Food Chem..

[8] Zhai H., Dong W., Fu X., Li G., Hu F. (2024). Integration of Widely Targeted Metabolomics and the E-Tongue Reveals the Chemical Variation and Taste Quality of Yunnan Arabica Coffee Prepared Using Different Primary Processing Methods. Food Chem. X.

[9] (2020). Green Coffee.

[10] Pękal A., Pyrzynska K. (2014). Evaluation of Aluminium Complexation Reaction for Flavonoid Content Assay. Food Anal. Methods.

[11] Bressani A.P.P., Batista N.N., Cassimiro D.M.d.J., Pires S.d.F., de Andrade H.M., Dias D.R., Schwan R.F. (2025). Exploring Coffee Processing Stages: Wet Fermentation with and without Saccharomyces Cerevisiae vs. Conventional Process. World J. Microbiol. Biotechnol..

[12] Dong W., Hu R., Long Y., Li H., Zhang Y., Zhu K., Chu Z. (2019). Comparative Evaluation of the Volatile Profiles and Taste Properties of Roasted Coffee Beans as Affected by Drying Method and Detected by Electronic Nose, Electronic Tongue, and HS-SPME-GC-MS. Food Chem..

[13] Tan Y., Wu H., Shi L., Barrow C., Dunshea F.R., Suleria H.A.R. (2023). Impacts of Fermentation on the Phenolic Composition, Antioxidant Potential, and Volatile Compounds Profile of Commercially Roasted Coffee Beans. Fermentation.

[14] Halagarda M., Obrok P. (2023). Influence of Post-Harvest Processing on Functional Properties of Coffee (*Coffea arabica* L.). Molecules.

[15] Haile M., Kang W.H. (2019). Antioxidant Activity, Total Polyphenol, Flavonoid and Tannin Contents of Fermented Green Coffee Beans with Selected Yeasts. Fermentation.

[16] Fujimoto H., Narita Y., Iwai K., Hanzawa T., Kobayashi T., Kakiuchi M., Ariki S., Wu X., Miyake K., Tahara Y. (2021). Bitterness Compounds in Coffee Brew Measured by Analytical Instruments and Taste Sensing System. Food Chem..

[17] Yeager S.E., Batali M.E., Guinard J.-X., Ristenpart W.D. (2023). Acids in Coffee: A Review of Sensory Measurements and Meta-Analysis of Chemical Composition. Crit. Rev. Food Sci. Nutr..

[18] Farah A., De Paulis T., Trugo L.C., Martin P.R. (2005). Effect of Roasting on the Formation of Chlorogenic Acid Lactones in Coffee. J. Agric. Food Chem..

[19] Rune C.J.B., Giacalone D., Steen I., Duelund L., Münchow M., Clausen M.P. (2023). Acids in Brewed Coffees: Chemical Composition and Sensory Threshold. Curr. Res. Food Sci..

[20] Martinez S.J., Bressani A.P.P., Dias D.R., Simão J.B.P., Schwan R.F. (2019). Effect of Bacterial and Yeast Starters on the Formation of Volatile and Organic Acid Compounds in Coffee Beans and Selection of Flavors Markers Precursors during Wet Fermentation. Front. Microbiol..

[21] Shen X., Zi C., Yang Y., Wang Q., Zhang Z., Shao J., Zhao P., Liu K., Li X., Fan J. (2023). Effects of Different Primary Processing Methods on the Flavor of Coffea Arabica Beans by Metabolomics. Fermentation.

[22] Bytof G., Knopp S.-E., Schieberle P., Teutsch I., Selmar D. (2005). Influence of Processing on the Generation of γ-Aminobutyric Acid in Green Coffee Beans. Eur. Food Res. Technol..

[23] de Melo Pereira G.V., de Carvalho Neto D.P., Magalhães Júnior A.I., Vásquez Z.S., Medeiros A.B.P., Vandenberghe L.P.S., Soccol C.R. (2019). Exploring the Impacts of Postharvest Processing on the Aroma Formation of Coffee Beans—A Review. Food Chem..

[24] Wang W., Zhou X., Liu Y. (2020). Characterization and Evaluation of Umami Taste: A Review. TrAC Trends Anal. Chem..

[25] Zhao C.J., Schieber A., Gänzle M.G. (2016). Formation of Taste-Active Amino Acids, Amino Acid Derivatives and Peptides in Food Fermentations—A Review. Food Res. Int..

[26] Shakoor A., Zhang C., Xie J., Yang X. (2022). Maillard Reaction Chemistry in Formation of Critical Intermediates and Flavour Compounds and Their Antioxidant Properties. Food Chem..

[27] Alcantara G.M.R.N., Martins L.C., Gomes W.P.C., Dresch D., Rocha F.R.P., Melchert W.R. (2025). Effect of Roasting on Chemical Composition of Coffee. Food Chem..

[28] Aswathi K.N., Shirke A., Praveen A., Chaudhari S.R., Murthy P.S. (2023). Pulped Natural/Honey Robusta Coffee Fermentation Metabolites, Physico-Chemical and Sensory Profiles. Food Chem..

[29] Shankar S.R., Sneha H.P., Prakash I., Khan M., Kumar P.H.N., Om H., Basavaraj K., Murthy P.S. (2022). Microbial Ecology and Functional Coffee Fermentation Dynamics with Pichia Kudriavzevii. Food Microbiol..

[30] Fang X., Xue R., Xiao J., Pu Q., Wang Y., Yuan Y., Liu B., Sui M., Jiang G., Niaz R. (2024). Effects of Different Fermentation Modes on Tea Leaves: Revealing the Metabolites Modification by Quasi-Targeted Metabolomics. Food Biosci..

[31] Wu H., Gu J., Bk A., Nawaz M.A., Barrow C.J., Dunshea F.R., Suleria H.A.R. (2022). Effect of Processing on Bioaccessibility and Bioavailability of Bioactive Compounds in Coffee Beans. Food Biosci..

[32] He M., Qin C.-X., Wang X., Ding N.-Z. (2020). Plant Unsaturated Fatty Acids: Biosynthesis and Regulation. Front. Plant Sci..

[33] Fakhrzad F., Jowkar A. (2024). Gene Expression Analysis of Drought Tolerance and Cuticular Wax Biosynthesis in Diploid and Tetraploid Induced Wallflowers. BMC Plant Biol..

[34] Wang Y., Wang X., Du P., Liu X., He S., Li L., Liu X., Chen Z. (2025). Lipidomic Profiling Provides Insights on Arabica Coffee Flavor Diversity in Different Postharvest Processing Methods. Curr. Res. Food Sci..

[35] Chagas Junior G.C.A., Ferreira N.R., Lopes A.S. (2021). The Microbiota Diversity Identified during the Cocoa Fermentation and the Benefits of the Starter Cultures Use: An Overview. Int. J. Food Sci. Technol..

[36] Shi Y., Li J., Zhou L., Zhang J., Feng X., Xing W., Tang C., Bai Y. (2025). Exploring the Contribution of Phosphatidylcholine and Triglyceride on the Formation of Beef Aroma-Active Compounds with Thermal Oxidation System. Curr. Res. Food Sci..

[37] Langfelder P., Horvath S. (2008). WGCNA: An R Package for Weighted Correlation Network Analysis. BMC Bioinf..

[38] He K., Peng X., Li Y., Zhao M., Feng Y. (2025). Revealing Metabolite Profiles in Soy Sauce and Exploring Their Correlation with Umami Taste Using UPLC-Orbitrap-MS/MS and GC-Tof-MS Derivatization. Food Chem..

[39] Breslin P.A.S. (1996). Interactions among salty, sour and bitter compounds. Trends Food Sci. Technol..

[40] Matteau J.-P., Chagnon P.-L., Célicourt P., Bonakdari H., Gumiere S.J. (2024). Chapter 1—Partial Least Squares Regression to Explore and Predict Environmental Data. Intelligence Systems for Earth, Environmental and Planetary Sciences.

[41] (2018). Methodology for Sensory Evaluation of Tea.

