# Supplementary material for: Analysis of Changes in Taste Characteristics of Coffee at Different Primary Processing Methods Using E-Tongue, Untargeted Metabolomics and WGCNA
Source: Foods. 2026 Apr 23;15(9):1475. doi: 10.3390/foods15091475 (PMC13163802; doi:10.3390/foods15091475)
Supplement: Supplementary file 1 [file foods-15-01475-s001.zip › foods-4230835-supplementary.pdf]

Analysis of changes in taste characteristics of coffee at different primary processing methods using E-tongue, untargeted metabolomics and WGCNA

Ying Liang[1], Yaqian Yuan[1], Jia Wang[1], Wenxue Chen[1], Weijun Chen[1], Qiuping Zhong[1], Jianfei Pei[1], Chun Chen[2], Xiong Fu[2], Rongrong He [1,*], Haiming Chen[1,3,*]

[1] Hainan Nongken Investment Holding Group Co., Ltd., Collaborative Innovation Laboratory, College of Food Sciences & Engineering, Hainan University, 58 People Road, Haikou 570228, China

[2] School of Food Science and Engineering, South China University of Technology, Guangzhou 510640, China

[3] Haikou Key Laboratory of Special Foods, Haikou 570228, China

* Correspondence: rongronghe@hainanu.edu.cn (R.H.); hmchen168@126.com (H.C.); Tel./Fax: +86-0898-66256495 (H.C.)

Figure S1. Radar graph of coffee samples sensory attributes.

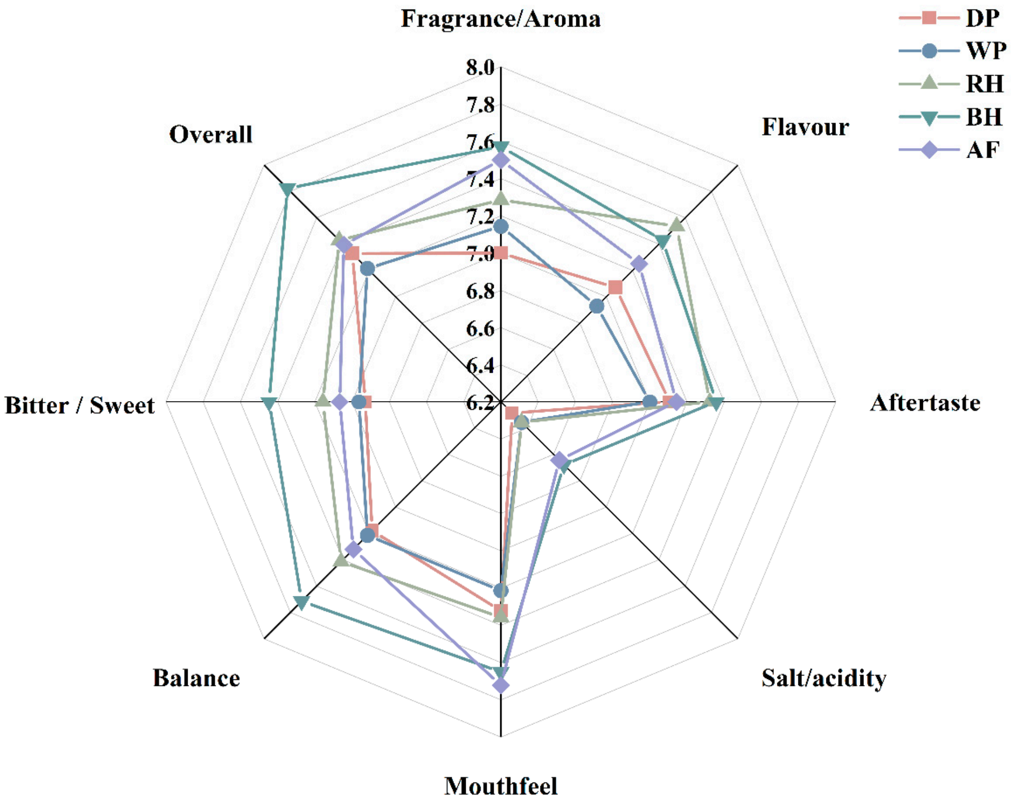

Figure S2. Multivariate analysis and hierarchical clustering of coffee samples based on primary processing methods. (A) OPLS-DA score plot showing distinct separation of the five groups with tight clustering of biological replicates. (B) Permutation test (200 permutations) confirming model robustness ($R^2Y = 0.998$, $Q^2 = 0.825$, $p < 0.005$). (C) Heatmap from hierarchical clustering of differential metabolites, highlighting clear metabolic differences between processing methods.

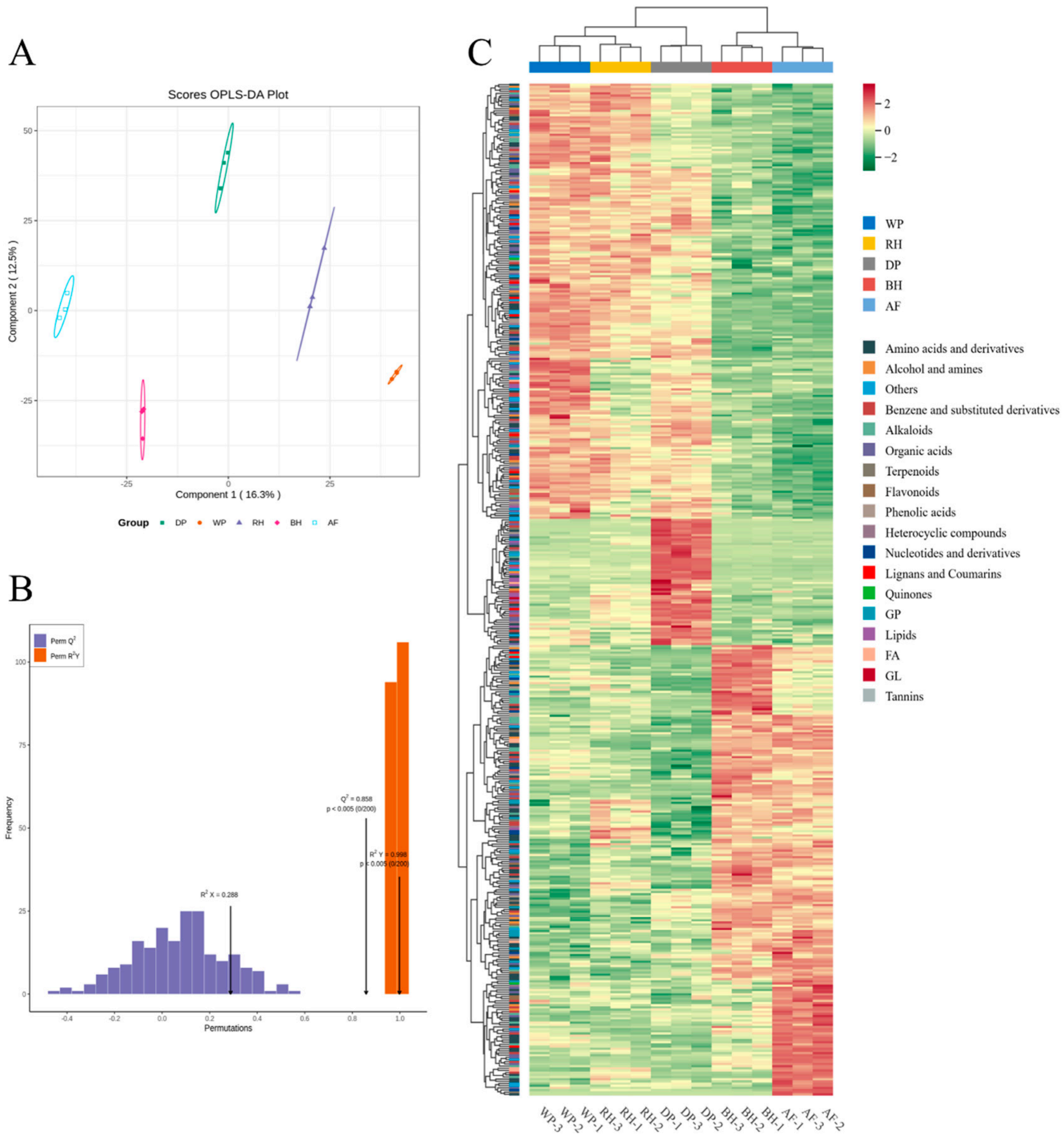

Figure S3 Validation and visualization of differential metabolites. (A) Model validation using the DP group as the reference. (B) Permutation test (200 runs) to confirm model robustness. (C) Volcano plot of differential metabolites (FC $\geq$ 2 or $\leq$ 0.5, VIP > 1, $p < 0.05$). (D) Bar chart of the top 20 differential metabolites based on fold change. (E) Pathway enrichment analysis of differential metabolites. (The order of paired comparisons from top to bottom is: WP vs DP, RH vs DP, BH vs DP, AF vs DP.)

A

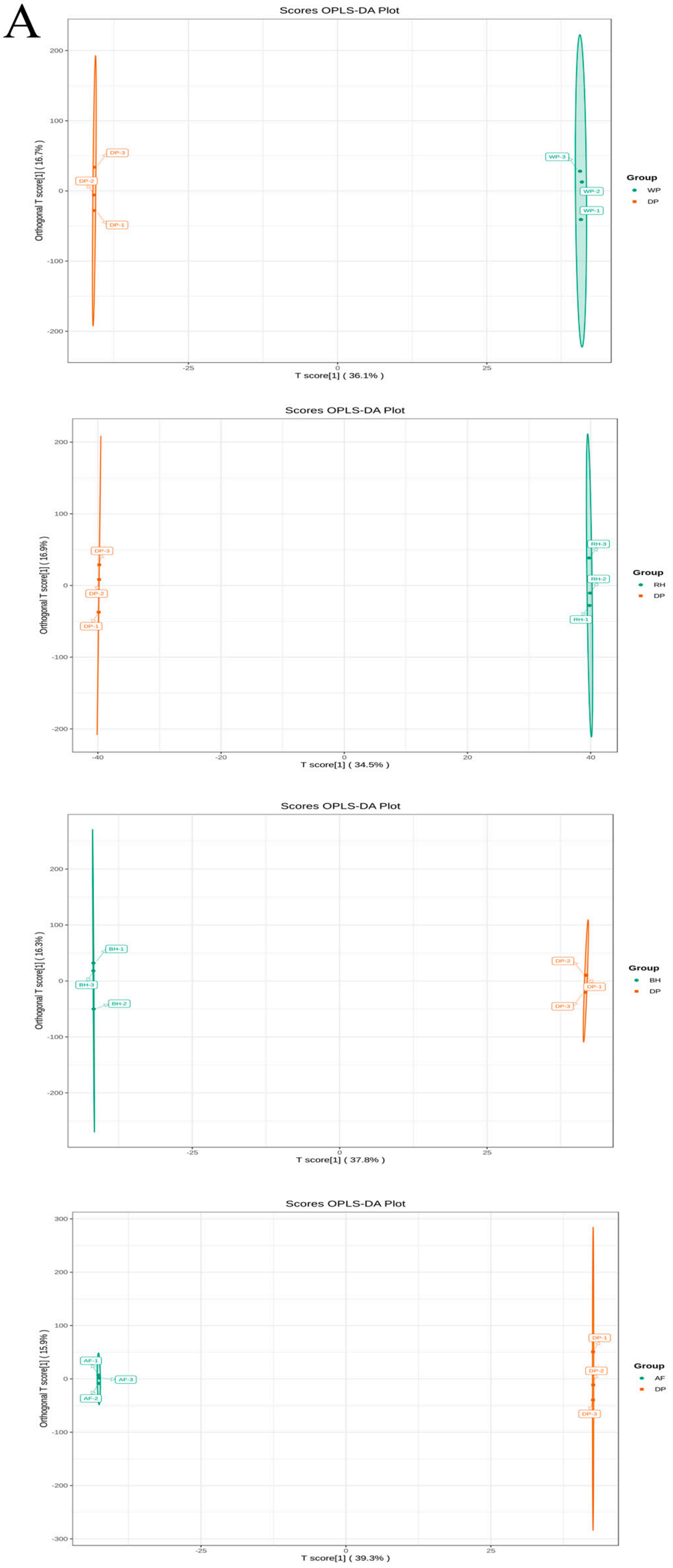
Scores OPLS-DA Plot
Orthogonal T score[1] (16.7%)
T score[1] (36.1%)
Group
WP
DP
Scores OPLS-DA Plot
Orthogonal T score[1] (16.9%)
T score[1] (34.5%)
Group
RH
DP
Scores OPLS-DA Plot
Orthogonal T score[1] (16.3%)
T score[1] (37.8%)
Group
BH
DP
Scores OPLS-DA Plot
Orthogonal T score[1] (15.9%)
T score[1] (39.3%)
Group
AF
DP

B

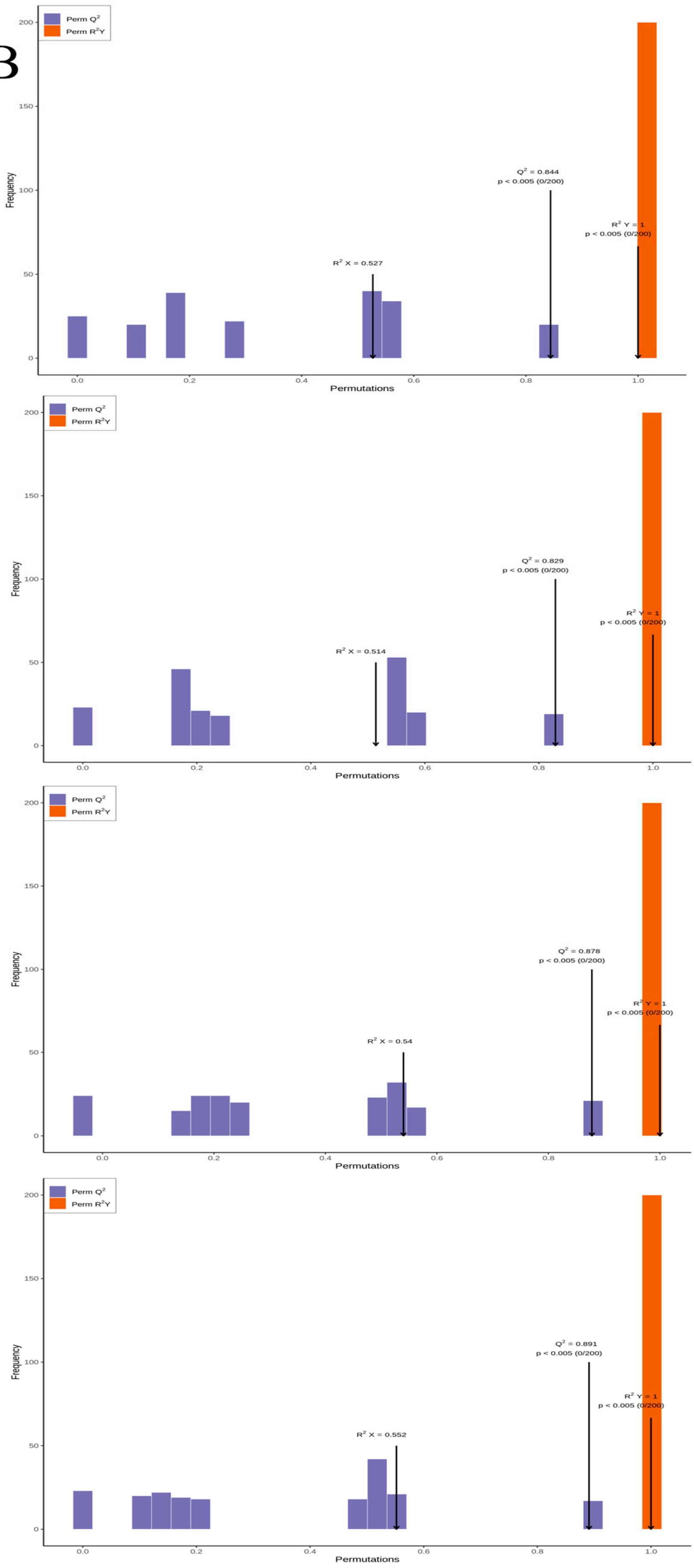
Perm Q2
Perm R2Y
Frequency
Permutations
Q2 = 0.844
p < 0.005 (0/200)
R2 Y = 1
p < 0.005 (0/200)
R2 X = 0.527
Q2 = 0.829
p < 0.005 (0/200)
R2 Y = 1
p < 0.005 (0/200)
R2 X = 0.514
Q2 = 0.878
p < 0.005 (0/200)
R2 Y = 1
p < 0.005 (0/200)
R2 X = 0.54
Q2 = 0.891
p < 0.005 (0/200)
R2 Y = 1
p < 0.005 (0/200)
R2 X = 0.552

C

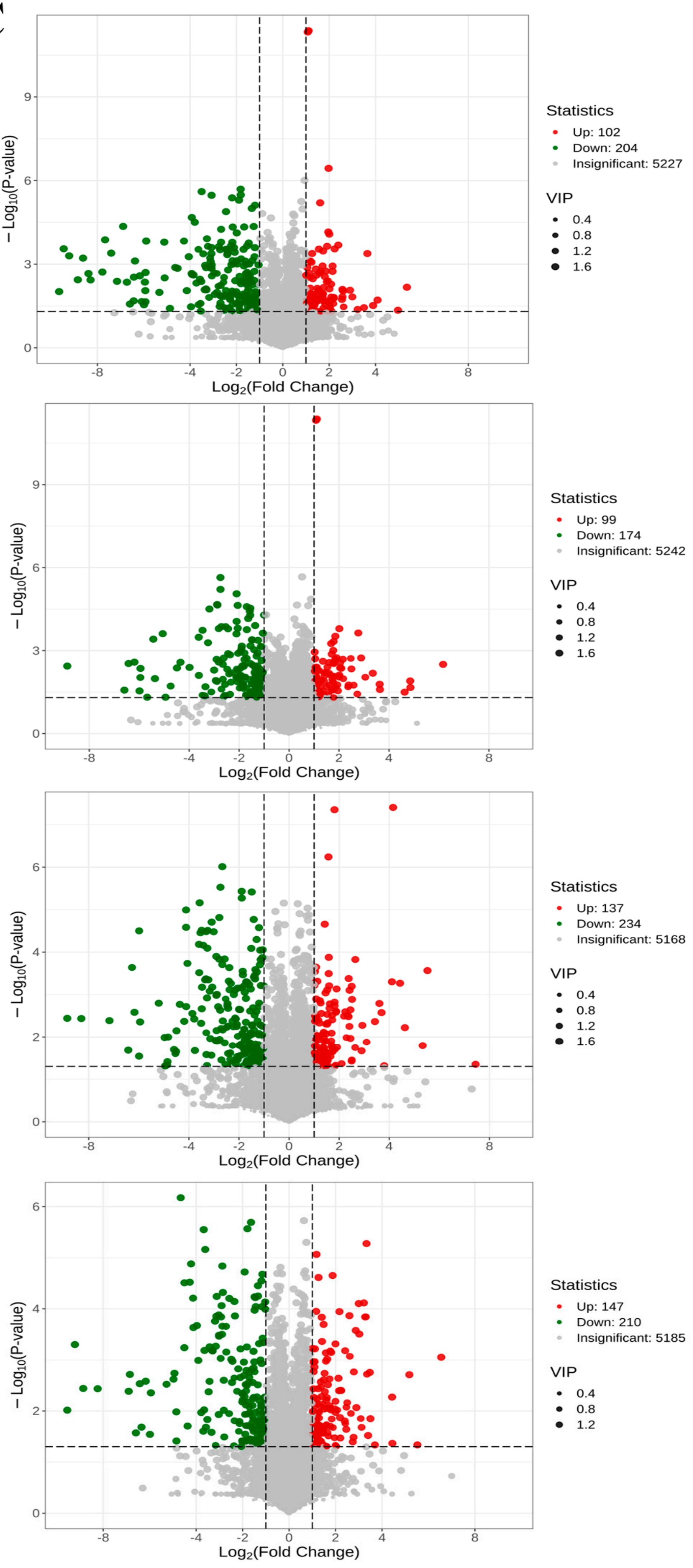

− Log10(P-value)
Log2(Fold Change)
Statistics
Up: 102
Down: 204
Insignificant: 5227
VIP
0.4
0.8
1.2
1.6
− Log10(P-value)
Log2(Fold Change)
Statistics
Up: 99
Down: 174
Insignificant: 5242
VIP
0.4
0.8
1.2
1.6
− Log10(P-value)
Log2(Fold Change)
Statistics
Up: 137
Down: 234
Insignificant: 5168
VIP
0.4
0.8
1.2
1.6
− Log10(P-value)
Log2(Fold Change)
Statistics
Up: 147
Down: 210
Insignificant: 5185
VIP
0.4
0.8
1.2

D

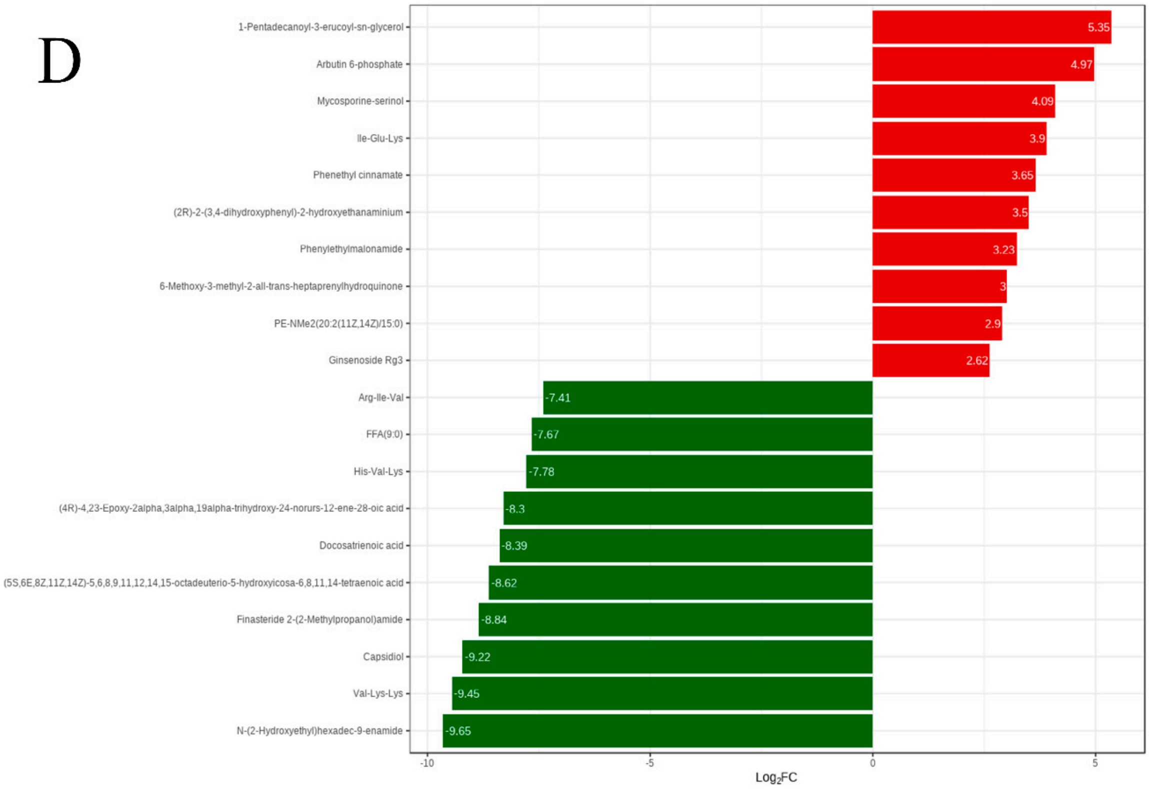

1-Pentadecanoyl-3-erucoyl-sn-glycerol
Arbutin 6-phosphate
Mycosporine-serinol
Ile-Glu-Lys
Phenethyl cinnamate
(2R)-2-(3,4-dihydroxyphenyl)-2-hydroxyethanaminium
Phenylethylmalonamide
6-Methoxy-3-methyl-2-all-trans-heptaprenylhydroquinone
PE-NMe2(20:2(11Z,14Z)/15:0)
Ginsenoside Rg3
Arg-Ile-Val
FFA(9:0)
His-Val-Lys
(4R)-4,23-Epoxy-2alpha,3alpha,19alpha-trihydroxy-24-norurs-12-ene-28-oic acid
Docosatrienoic acid
(5S,6E,8Z,11Z,14Z)-5,6,8,9,11,12,14,15-octadeuterio-5-hydroxyicosa-6,8,11,14-tetraenoic acid
Finasteride 2-(2-Methylpropanol)amide
Capsidiol
Val-Lys-Lys
N-(2-Hydroxyethyl)hexadec-9-enamide
5.35
4.97
4.09
3.9
3.65
3.5
3.23
3
2.9
2.62
-7.41
-7.67
-7.78
-8.3
-8.39
-8.62
-8.84
-9.22
-9.45
-9.65
-10
-5
0
5
Log2FC

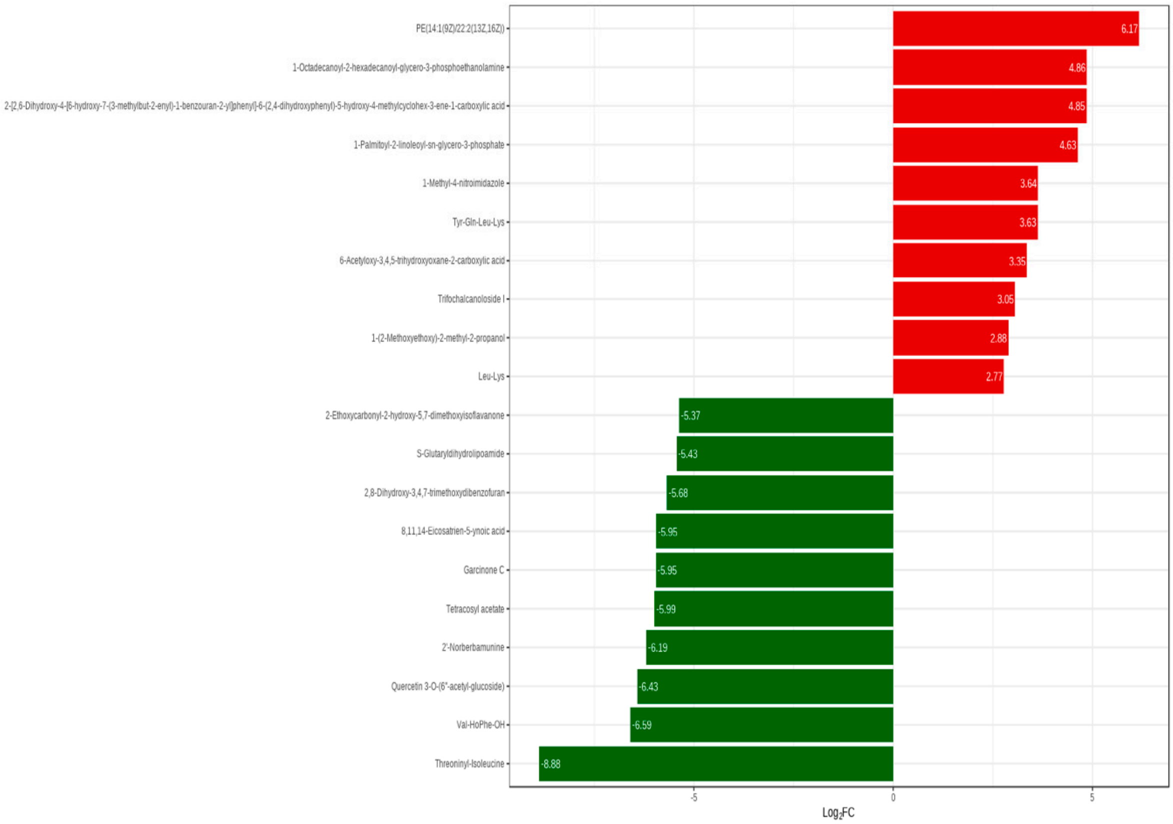

PE(14:1(9Z)/22:2(13Z,16Z))
1-Octadecanoyl-2-hexadecanoyl-glycero-3-phosphoethanolamine
2-[2,6-Dihydroxy-4-[6-hydroxy-7-(3-methylbut-2-enyl)-1-benzouran-2-yl]phenyl]-6-(2,4-dihydroxyphenyl)-5-hydroxy-4-methylcyclohex-3-ene-1-carboxylic acid
1-Palmitoyl-2-linoleoyl-sn-glycero-3-phosphate
1-Methyl-4-nitroimidazole
Tyr-Gln-Leu-Lys
6-Acetyloxy-3,4,5-trihydroxyoxane-2-carboxylic acid
Trifochalcanoloside I
1-(2-Methoxyethoxy)-2-methyl-2-propanol
Leu-Lys
2-Ethoxycarbonyl-2-hydroxy-5,7-dimethoxyisoflavanone
S-Glutaryldihydrolipoamide
2,8-Dihydroxy-3,4,7-trimethoxydibenzofuran
8,11,14-Eicosatrien-5-ynoic acid
Garcinone C
Tetracosyl acetate
2'-Norberbamunine
Quercetin 3-O-(6"-acetyl-glucoside)
Val-HoPhe-OH
Threoninyl-Isoleucine
6.17
4.86
4.85
4.63
3.64
3.63
3.35
3.05
2.88
2.77
-5.37
-5.43
-5.68
-5.95
-5.95
-5.99
-6.19
-6.43
-6.59
-8.88
-5
0
5
Log2FC

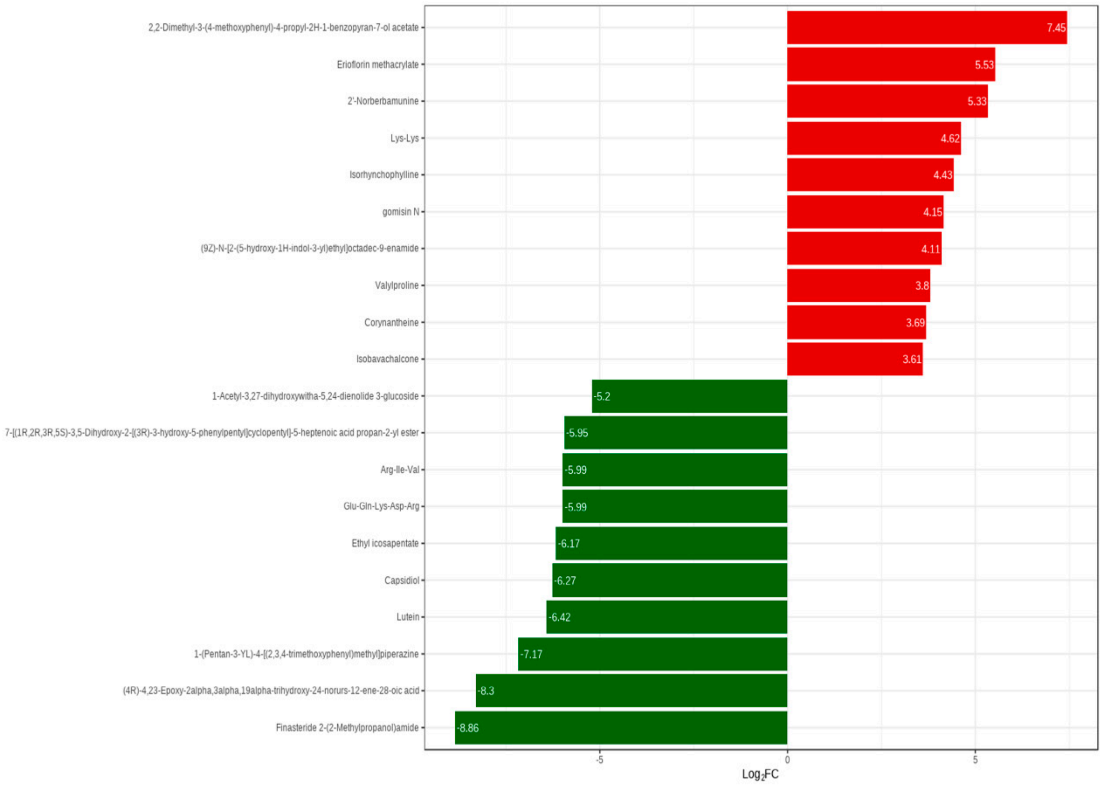
2,2-Dimethyl-3-(4-methoxyphenyl)-4-propyl-2H-1-benzopyran-7-ol acetate
Erioflorin methacrylate
2'-Norberbamunine
Lys-Lys
Isorhynchophylline
gomisin N
(9Z)-N-[2-(5-hydroxy-1H-indol-3-yl)ethyl]octadec-9-enamide
Valylproline
Corynantheine
Isobavachalcone
1-Acetyl-3,27-dihydroxywitha-5,24-dienolide 3-glucoside
7-[(1R,2R,3R,5S)-3,5-Dihydroxy-2-[(3R)-3-hydroxy-5-phenylpentyl]cyclopentyl]-5-heptenoic acid propan-2-yl ester
Arg-Ile-Val
Glu-Gln-Lys-Asp-Arg
Ethyl icosapentate
Capsidiol
Lutein
1-(Pentan-3-YL)-4-[(2,3,4-trimethoxyphenyl)methyl]piperazine
(4R)-4,23-Epoxy-2alpha,3alpha,19alpha-trihydroxy-24-norurs-12-ene-28-oic acid
Finasteride 2-(2-Methylpropanol)amide
7.45
5.53
5.33
4.62
4.43
4.15
4.11
3.8
3.69
3.61
-5.2
-5.95
-5.99
-5.99
-6.17
-6.27
-6.42
-7.17
-8.3
-8.86
-5
0
5
Log2FC

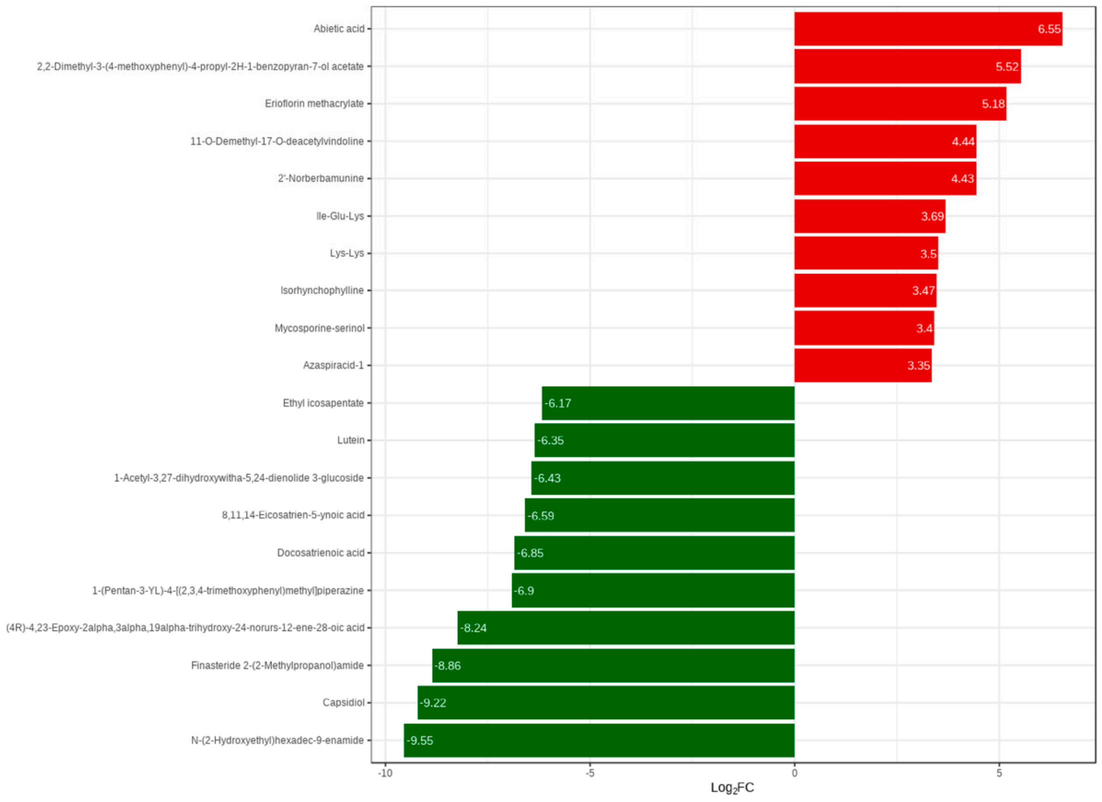
Abietic acid
2,2-Dimethyl-3-(4-methoxyphenyl)-4-propyl-2H-1-benzopyran-7-ol acetate
Erioflorin methacrylate
11-O-Demethyl-17-O-deacetylvindoline
2'-Norberbamunine
Ile-Glu-Lys
Lys-Lys
Isorhynchophylline
Mycosporine-serinol
Azaspiracid-1
Ethyl icosapentate
Lutein
1-Acetyl-3,27-dihydroxywitha-5,24-dienolide 3-glucoside
8,11,14-Eicosatrien-5-ynoic acid
Docosatrienoic acid
1-(Pentan-3-YL)-4-[(2,3,4-trimethoxyphenyl)methyl]piperazine
(4R)-4,23-Epoxy-2alpha,3alpha,19alpha-trihydroxy-24-norurs-12-ene-28-oic acid
Finasteride 2-(2-Methylpropanol)amide
Capsidiol
N-(2-Hydroxyethyl)hexadec-9-enamide
6.55
5.52
5.18
4.44
4.43
3.69
3.5
3.47
3.4
3.35
-6.17
-6.35
-6.43
-6.59
-6.85
-6.9
-8.24
-8.86
-9.22
-9.55
-10
-5
0
5
Log2FC

E

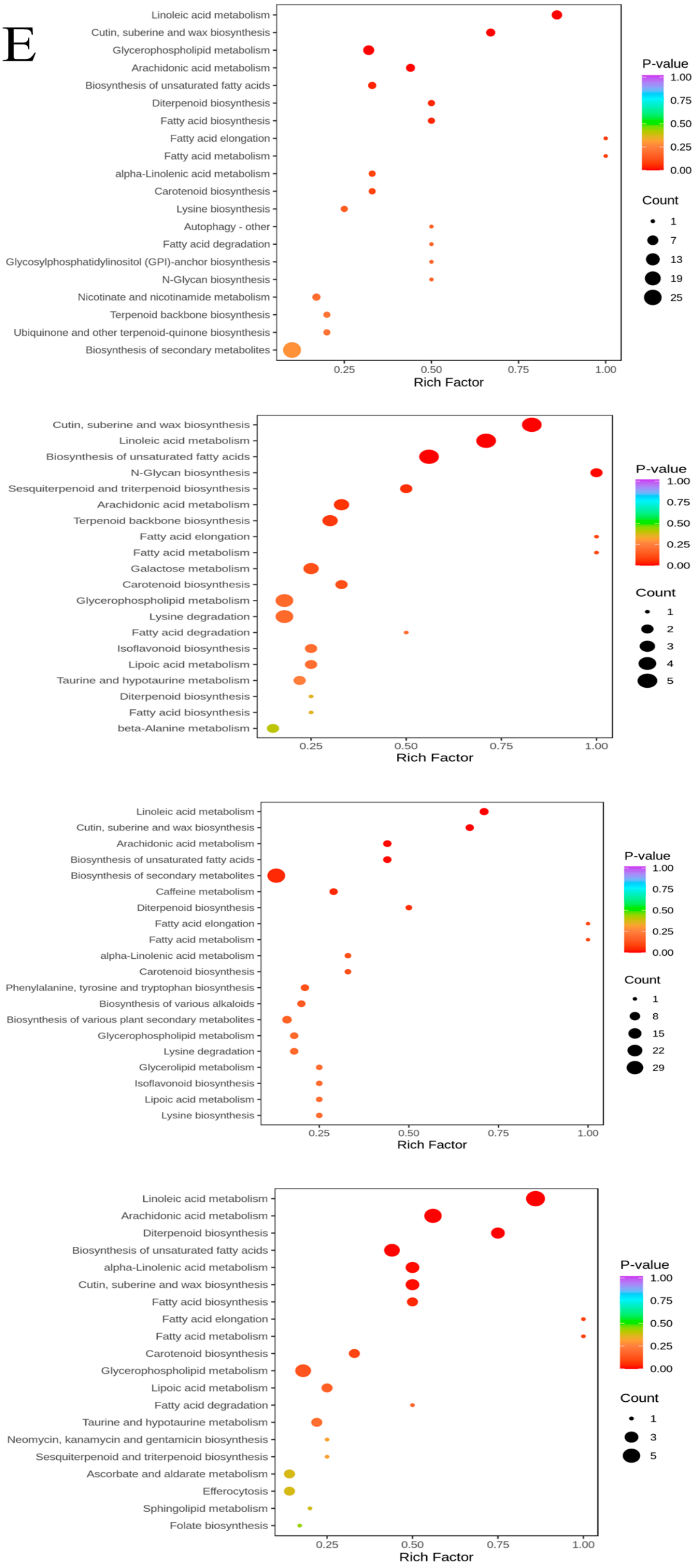
Linoleic acid metabolism
Cutin, suberine and wax biosynthesis
Glycerophospholipid metabolism
Arachidonic acid metabolism
Biosynthesis of unsaturated fatty acids
Diterpenoid biosynthesis
Fatty acid biosynthesis
Fatty acid elongation
Fatty acid metabolism
alpha-Linolenic acid metabolism
Carotenoid biosynthesis
Lysine biosynthesis
Autophagy - other
Fatty acid degradation
Glycosylphosphatidylinositol (GPI)-anchor biosynthesis
N-Glycan biosynthesis
Nicotinate and nicotinamide metabolism
Terpenoid backbone biosynthesis
Ubiquinone and other terpenoid-quinone biosynthesis
Biosynthesis of secondary metabolites
0.25
0.50
0.75
1.00
Rich Factor
P-value
1.00
0.75
0.50
0.25
0.00
Count
1
7
13
19
25
Cutin, suberine and wax biosynthesis
Linoleic acid metabolism
Biosynthesis of unsaturated fatty acids
N-Glycan biosynthesis
Sesquiterpenoid and triterpenoid biosynthesis
Arachidonic acid metabolism
Terpenoid backbone biosynthesis
Fatty acid elongation
Fatty acid metabolism
Galactose metabolism
Carotenoid biosynthesis
Glycerophospholipid metabolism
Lysine degradation
Fatty acid degradation
Isoflavonoid biosynthesis
Lipoic acid metabolism
Taurine and hypotaurine metabolism
Diterpenoid biosynthesis
Fatty acid biosynthesis
beta-Alanine metabolism
0.25
0.50
0.75
1.00
Rich Factor
P-value
1.00
0.75
0.50
0.25
0.00
Count
1
2
3
4
5
Linoleic acid metabolism
Cutin, suberine and wax biosynthesis
Arachidonic acid metabolism
Biosynthesis of unsaturated fatty acids
Biosynthesis of secondary metabolites
Caffeine metabolism
Diterpenoid biosynthesis
Fatty acid elongation
Fatty acid metabolism
alpha-Linolenic acid metabolism
Carotenoid biosynthesis
Phenylalanine, tyrosine and tryptophan biosynthesis
Biosynthesis of various alkaloids
Biosynthesis of various plant secondary metabolites
Glycerophospholipid metabolism
Lysine degradation
Glycerolipid metabolism
Isoflavonoid biosynthesis
Lipoic acid metabolism
Lysine biosynthesis
0.25
0.50
0.75
1.00
Rich Factor
P-value
1.00
0.75
0.50
0.25
0.00
Count
1
8
15
22
29
Linoleic acid metabolism
Arachidonic acid metabolism
Diterpenoid biosynthesis
Biosynthesis of unsaturated fatty acids
alpha-Linolenic acid metabolism
Cutin, suberine and wax biosynthesis
Fatty acid biosynthesis
Fatty acid elongation
Fatty acid metabolism
Carotenoid biosynthesis
Glycerophospholipid metabolism
Lipoic acid metabolism
Fatty acid degradation
Taurine and hypotaurine metabolism
Neomycin, kanamycin and gentamicin biosynthesis
Sesquiterpenoid and triterpenoid biosynthesis
Ascorbate and aldarate metabolism
Efferocytosis
Sphingolipid metabolism
Folate biosynthesis
0.25
0.50
0.75
1.00
Rich Factor
P-value
1.00
0.75
0.50
0.25
0.00
Count
1
3
5

We verified the validity of the models using the DP group as a reference (Fig. S3A) and conducted 200-run permutation tests to confirm it (Fig. S3B). The differential metabolites (FC ≥ 2 or ≤ 0.5, variable importance in projection (VIP) > 1, and $p < 0.05$) were visualized using volcano plots (Fig. S3C), Top20 FC bar chart (Fig. S3D) and pathway enrichment maps (Fig. S3E). The samples exhibited clear separation along the positive and negative sides of t [1], indicating substantial compositional divergence. Permutation results (R^2Y, $Q^2 > 0.7$, $p < 0.005$) confirmed the model's strong performance.

Volcano plots were used to visualize intergroup metabolic differences (Fig. S3C), and Venn diagrams were used to summarize the overlaps among comparisons (Fig. S4). In every pairwise contrast, the number of down-regulated metabolites exceeded the number of up-regulated ones. A total of 371 differential metabolites were identified between DP and BH: 234 were higher in DP, and 137 were higher in BH. Similarly, a comparison between DP and AF revealed 357 metabolites, with 210 being higher in DP and 147 in AF. The DP-WP and DP-RH comparisons yielded 306 and 273 differential metabolites, respectively; DP-RH demonstrated the least variation. These trends align with the PCA distribution, confirming pronounced metabolic divergence among the various processing treatments.

The 20 most significantly altered metabolites (10 up-regulated and 10 down-regulated) were selected for a more detailed comparison (Fig. S3D). The up-regulated compounds in WP, RH, DP, and AF were primarily lipids, amino acid derivatives, and

terpenoids. Key lipids identified in this study include ethyl icosapentate, docosatrienoic acid, N-methyl phosphatidylethanolamine (14:1/16:1), phosphatidylethanolamine (14:1/22:2), and 1-octadecanoyl-2-hexadecanoyl-PE.

Polyunsaturated fatty acids (PUFAs) and esters are susceptible to enzymatic or auto-oxidation, resulting in the production of volatile C_6-C_{10} aldehydes and ketones, such as 2,4-decadienal and hexanal. These compounds contribute to herbal, oily, and nutty notes (Hammer & Schieberle, 2013; Wang et al., 2023). Long-chain PUFAs, such as ethyl pentacosan-5-enoate and docosatrienoic acid, readily oxidize, forming aldehydes known to contribute to the aroma of baked and roasted foods. Furthermore, lipid-derived aldehydes undergo Strecker degradation with amino acids, linking lipid oxidation to the Maillard reaction and enriching volatile diversity (Adams et al., 2011). Additionally, phospholipids act as oxidation precursors and regulators of volatile release at interfaces, influencing aroma intensity and persistence (Li et al., 2024). Specifically, N-methyl phosphatidylethanolamine (14:1/16:1) and phosphatidylethanolamine (14:1/22:2) modulate the distribution of oxidation intermediates and Maillard products, enhancing the continuity of fatty and roasted notes.

Up-regulated amino acids and peptides, including phenylalanine-tyrosine-methionine, valine-alanine, lysine-lysine, and isoleucine-glutamic acid-lysine, have been identified as flavor-active compounds. These compounds act as nitrogen donors in Maillard and Strecker reactions, fostering flavors reminiscent of nuts and roasting (de Sousa Fontes et al., 2024; Zhang et al., 2023).

Conversely, down-regulated metabolites were predominantly lipids, sterols, flavonoids, and glycosides. The observed decline in their overall abundance is attributed to structural disruption of bean tissues during processing. This disruption has been shown to result in reduced metabolic activity and subsequent down-regulation of primary metabolites.

Figure S4 Venn diagram of differential metabolites among pairwise comparisons.

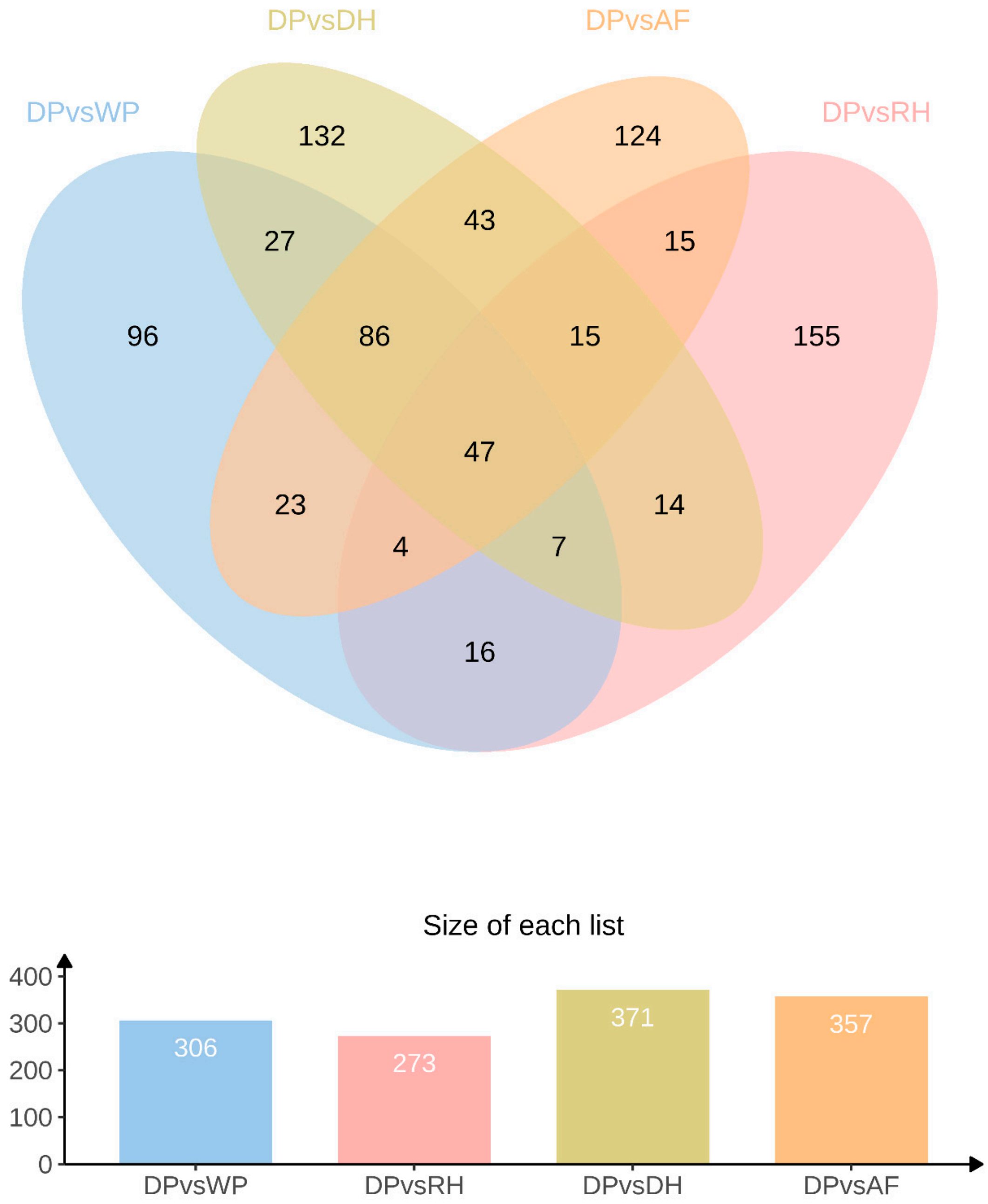

Figure S5. Heatmap using key differential metabolites identified by targeted metabolomic and WGCNA analyses as the input matrix. (A) Amino acids and derivatives (n =107). (B) Organic acids (n = 62). (C) Benzene and its derivatives (n = 60). (D) Alkaloids (n = 30). (E) Phenolic acids(n=27).

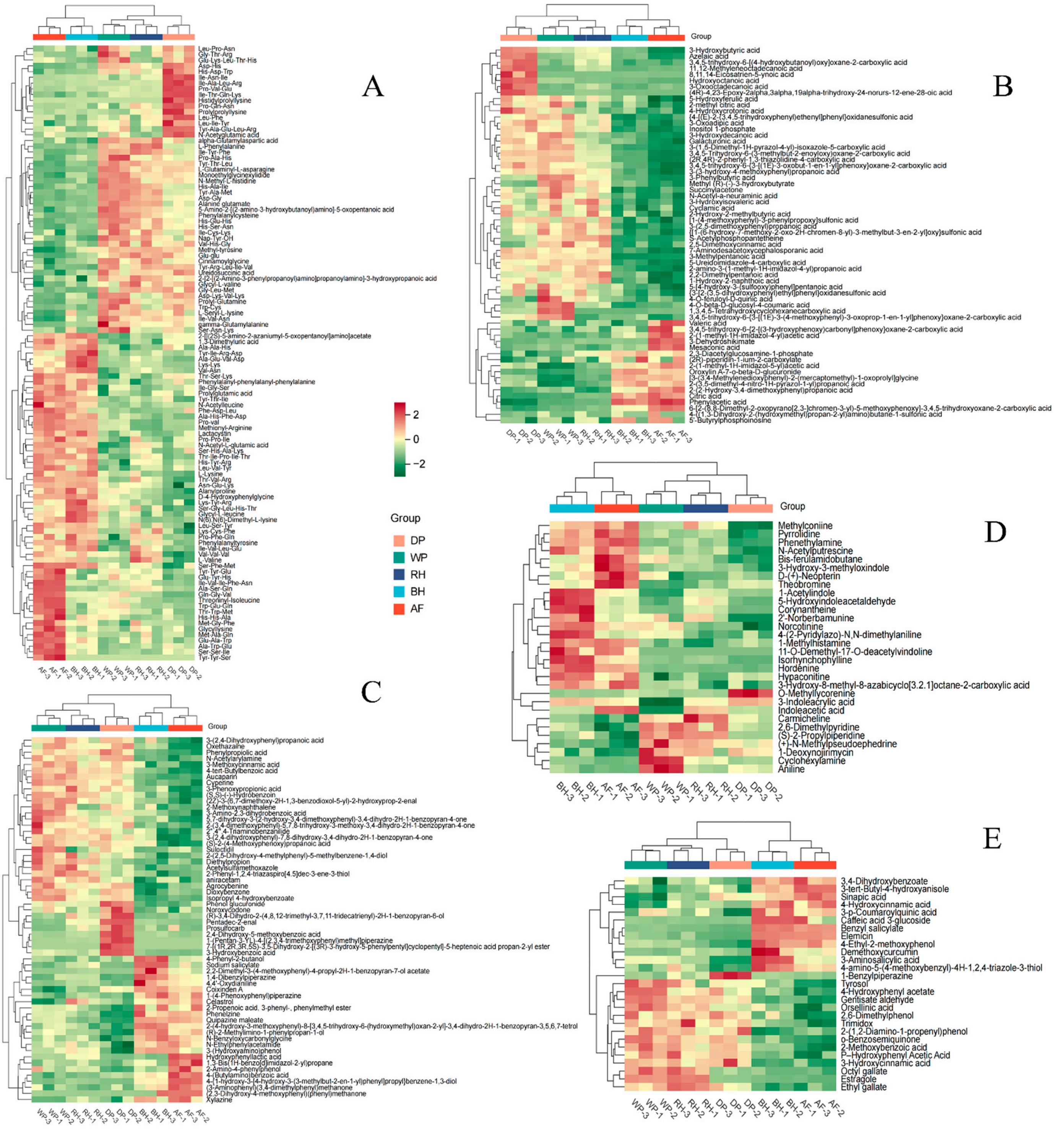

a. Amino acids and derivatives

During roasting, certain amino acids act as aroma precursors that contribute substantially to the overall flavor of the brewed coffee (Yeager et al., 2023). Heatmap analysis (Fig. S5A) revealed distinct bidirectional changes in amino acids and derivatives across treatments. Compared with DP, AF and BH revealed marked increases in aromatic and sulfur-containing short peptides such as Ser-Phe-Met, Ala-His-Phe-Asp, whereas several branched-chain amino acids-related peptides, including Leu-Pro-Asn and Ile-Thr-Gln-Lys, were decreased. The RH group exhibited relatively minor alterations, while WP showed higher levels of some aromatic peptides such as Tyr-Ile-Arg-Asp. Aromatic and sulfur-containing amino acids participate in the Maillard and Strecker reactions, yielding pyrazines and thiophenes that enhance roasted and nutty notes (Maoz et al., 2022). Variations in amino acid content also affect taste perception, where reductions in branched-chain amino acids decrease the intensity of bitterness (Chu et al., 2024; Miyanaga et al., 2002). Increased aromatic amino acids can form "kokumi" peptides through γ-glutamylation, enhancing palatability and reducing bitterness (Yang et al., 2019).

b. Organic acids

Organic acids are critical determinants of coffee acidity and mouthfeel, contributing fruity or citrus-like notes (Santanatoglia et al., 2024) and moderating bitterness through taste interactions (Keast & Breslin, 2003). Heatmap results (Fig. S5B) revealed notable changes in the composition of organic acids among the different treatments. Compared

to DP, both BH and AF exhibited overall declines, except for phenylacetic and citric acids, which increased significantly ($p < 0.05$). Phenylacetic acid increased 9.23- and 13.67-fold and citric acid increased 2.02- and 2.30-fold, respectively. Phenylacetic acid imparts a honey-like aroma, and citric acid enhances bright, fruity acidity and balances bitterness (Junge et al., 2020; Rune et al., 2023; Sun et al., 2024).

In contrast, the WP and RH contained higher overall organic acid levels. In WP, 1,3,4,5-tetrahydroxycyclohexanecarboxylic acid and 4-O-feruloyl-D-quinic acid increased significantly ($p < 0.05$), by 3.93- and 2.50-fold, respectively. These compounds are associated with enhanced bitterness (Asamenew et al., 2019; Frank et al., 2006), which is consistent with electronic-tongue results showing the strongest bitterness in the WP.

c. Benzene and substituted derivatives

Benzene and substituted derivatives also play a significant role in coffee flavor formation. The heatmap (Fig. S5C) reveals that different treatments exerted a pronounced effect on the distribution of Benzene and substituted derivatives content. Compared to the DP (control), WP and RH exhibited an overall upward trend, with most compounds showing higher relative abundance in these two samples. Conversely, BH and AF clustered closely together, exhibiting relatively lower overall content. This result contrasts with the trends observed in the organic acid analysis, indicating distinct metabolic pathway responses across different treatments.

Among significantly altered metabolites, 4-phenyl-2-butanol and Sodium salicylate were significantly up-regulated ($p < 0.05$) in the AF and BH, respectively, showing 2.49-fold and 2.97-fold increases compared to the DP. According to the good scents company information system, 4-phenyl-2-butanol possesses "floral, peony leaf, sweet, mimosa, and hyacinth-like" odors, along with "floral, spicy, magnolia, mango, green petals, and tropical melon-like" flavor characteristics. Its upregulation may contribute to enhanced floral and fruity notes in AF. Meanwhile, sodium salicylate is reported to mitigate bitterness (Vraneš et al., 2022), and its significant increase in concentration may correlate with the reduced bitterness observed in the BH.

d. Alkaloids

Alkaloids participate in defense, signal regulation, and bitter compound production during plant metabolism, significantly influencing the overall metabolic profile and sensory attributes of samples. Heatmap (Fig. S5D) reveal that different treatments significantly altered the distribution of alkaloid content.

Compared to the DP, certain biogenic amines were markedly upregulated in both BH and AF. Specifically, phenethylamine and hordenine showed significant upregulation in both groups ($p < 0.05$). The former is a key intermediate in aromatic amine metabolism and is associated with phenylalanine decarboxylation (Tieman et al., 2006); the latter belongs to the barley alkaloid family, regulating cellular signaling pathways and linked to stress responses (Ishiai et al., 2016). This suggests enhanced activity in the amino acid decarboxylation pathway (Visciano & Schirone, 2022).

In contrast, most indole and tryptophan derivatives were lower in WP and RH than in DP. Specifically, indoleacetic acid (IAA) and 3-indoleacrylic acid (3-IAAcr) were significantly downregulated in WP ($p < 0.05$), indicating reduced activity in the tryptophan degradation pathway or a shift in metabolic flux (Tang et al., 2023; Duca & Glick, 2020). Notably, cyclohexylamine, described in authoritative toxicology documents as having a "strong bitter taste," was significantly upregulated in WP, potentially contributing to the marked increase in bitterness observed in this group. This finding aligns with E-tongue results indicating WP as the most bitter among the five sample groups.

e. Phenolic acids

Phenolic acids and their related aromatic acid/aromatic phenol derivatives serve dual roles in flavor and physiological functions within plants and coffee processing systems. On one hand, they directly shape bitterness and astringency (Linne et al., 2025; Kraehenbuehl et al., 2017), while also serving as precursors for volatile phenolics and balsamic molecules, contributing to the formation and amplification of spicy, floral, and woody aromas (Debona et al., 2025; Tan et al., 2023). The phenolic acid heatmap (Fig. S5E) reveals that different primary processing methods significantly altered the abundance distribution of phenolic acid compounds, with samples forming distinct clusters based on treatment, indicating that processing techniques exert a significant regulatory effect on related metabolic pathways.

Compared to the DP, WP and RH exhibited an upward trend in multiple phenolic acids and aromatic acids associated with “fresh, floral, and slightly sweet” profiles, notably including estragole and tyrosol. Estragole is linked to fresh anise and green floral notes, while tyrosol contributes mild floral-fruity aromas (the good scents company information system). Their upregulation may contribute to enhanced brightness, cleanliness, and layered aroma perception in the cup profile. Conversely, the upregulation of 4-ethyl-2-methoxyphenol and elemicin in the BH and AF groups promotes woody and spicy notes, while benzyl salicylate, characterized by balsamic and floral qualities, may help mitigate excessive bitterness.

References

Adams, A., Kitrytė, V., Venskutonis, R., & De Kimpe, N. (2011). Model studies on the pattern of volatiles generated in mixtures of amino acids, lipid-oxidation-derived aldehydes, and glucose. *Journal of Agricultural and Food Chemistry*, *59*(4), 1449–1456. https://doi.org/10.1021/jf104091p

Asamenew, G., Kim, H.-W., Lee, M.-K., Lee, S.-H., Lee, S., Cha, Y.-S., Lee, S. H., Yoo, S. M., & Kim, J.-B. (2019). Comprehensive characterization of hydroxycinnamoyl derivatives in green and roasted coffee beans: A new group of methyl hydroxycinnamoyl quinate. *Food Chemistry: X*, *2*, 100033. https://doi.org/10.1016/j.fochx.2019.100033

Chu, X., Zhu, W., Li, X., Su, E., & Wang, J. (2024). Bitter flavors and bitter compounds in foods: Identification, perception, and reduction techniques. *Food Research International*, *183*, 114234. https://doi.org/10.1016/j.foodres.2024.114234

de Sousa Fontes, V. M., de Sousa Galvão, M., Moreira de Carvalho, L., do Nascimento Guedes, F. L., dos Santos Lima, M., Alencar Bezerra, T. K., & Madruga, M. S. (2024). Thiamine, cysteine and xylose added to the maillard reaction of goat protein hydrolysate potentiates the formation of meat flavoring compounds. *Food Chemistry*, *445*, 138398. https://doi.org/10.1016/j.foodchem.2024.138398

Debona, D. G., Lyrio, M. V. V., da Luz, J. M. R., Frinhani, R. Q., Araújo, B. Q., Oliveira, E. C. da S., Agnoletti, B. Z., Coura, M. R., Pereira, L. L., & de Castro, E. V. R. (2025). Comprehensive evaluation of volatile compounds and sensory profiles of coffee throughout the roasting process. *Food Chemistry*, *478*, 143586. https://doi.org/10.1016/j.foodchem.2025.143586

Duca, D. R., & Glick, B. R. (2020). Indole-3-acetic acid biosynthesis and its regulation in plant-associated bacteria. *Applied Microbiology and Biotechnology*, *104*(20), 8607–8619. https://doi.org/10.1007/s00253-020-10869-5

Frank, O., Zehentbauer, G., & Hofmann, T. (2006). Bioresponse-guided decomposition of roast coffee beverage and identification of key bitter taste compounds. *European Food Research and Technology*, *222*(5–6), 492–508. https://doi.org/10.1007/s00217-005-0143-6

Hammer, M., & Schieberle, P. (2013). Model studies on the key aroma compounds formed by an oxidative degradation of ω-3 fatty acids initiated by either copper(II) ions or lipoxygenase. *Journal of Agricultural and Food Chemistry*, *61*(46), 10891–10900. https://doi.org/10.1021/jf403827p

Ishiai, S., Kondo, H., Hattori, T., Mikami, M., Aoki, Y., Enoki, S., & Suzuki, S. (2016). Hordenine is responsible for plant defense response through jasmonate-dependent defense pathway. *Physiological and Molecular Plant Pathology*, *96*, 94–100. https://doi.org/10.1016/j.pmpp.2016.10.003

Junge, J. Y., Bertelsen, A. S., Mielby, L. A., Zeng, Y., Sun, Y.-X., Byrne, D. V., & Kidmose, U. (2020). Taste interactions between sweetness of sucrose and sourness of citric and tartaric acid among chinese and danish consumers. *Foods*, *9*(10), 1425. https://doi.org/10.3390/foods9101425

Keast, R. S. J., & Breslin, P. A. S. (2003). An overview of binary taste–taste interactions. *Food Quality and Preference*, *14*(2), 111–124. https://doi.org/10.1016/S0950-3293(02)00110-6

Kraehenbuehl, K., Page-Zoerkler, N., Gartenmann, K., & Blank, I. (2017). Selective enzymatic hydrolysis of chlorogenic acid lactones in a model system and in a coffee extract. Application to reduction of coffee bitterness. *Food Chemistry*, *218*, 9–14. https://doi.org/10.1016/j.foodchem.2016.09.055

Li, X., Zhao, Z., Shi, S., Li, D., Sang, Y., Wang, P., Zhao, L., Wang, F., Fang, B., Chen, S., Li, Y., Jiang, Z., Luo, J., Zhang, X., & Wang, R. (2024). Flavor properties of post-heated fermented milk revealed by a comprehensive analysis based on volatile and non-volatile metabolites and sensory evaluation. *Current Research in Food Science*, *9*, 100892. https://doi.org/10.1016/j.crfs.2024.100892

Linne, B. M., Tello, E., Simons, C. T., & Peterson, D. G. (2025). Chemical characterization and sensory evaluation of a phenolic-rich melanoidin isolate contributing to coffee astringency. *Food & Function*, *16*(7), 2870–2880. https://doi.org/10.1039/D4FO04934A

Maoz, I., Lewinsohn, E., & Gonda, I. (2022). Amino acids metabolism as a source for aroma volatiles biosynthesis. *Current Opinion in Plant Biology*, *67*, 102221. https://doi.org/10.1016/j.pbi.2022.102221

Miyanaga, Y., Tanigake, A., Nakamura, T., Kobayashi, Y., Ikezaki, H., Taniguchi, A., Matsuyama, K., & Uchida, T. (2002). Prediction of the bitterness of single, binary- and multiple-component amino acid solutions using a taste sensor. *International Journal of Pharmaceutics*, *248*(1), 207–218. https://doi.org/10.1016/S0378-5173(02)00456-8

Rune, C. J. B., Giacalone, D., Steen, I., Duelund, L., Münchow, M., & Clausen, M. P. (2023). Acids in brewed coffees: Chemical composition and sensory threshold. *Current Research in Food Science*, *6*, 100485. https://doi.org/10.1016/j.crfs.2023.100485

Santanatoglia, A., Angeloni, S., Caprioli, G., Fioretti, L., Ricciutelli, M., Vittori, S., & Alessandroni, L. (2024). Comprehensive investigation of coffee acidity on eight different brewing methods through chemical analyses, sensory evaluation and statistical elaboration. *Food Chemistry*, *454*, 139717. https://doi.org/10.1016/j.foodchem.2024.139717

Sun, Z., Lin, Y., Yang, H., Zhao, R., Zhu, J., & Wang, F. (2024). Characterization of honey-like characteristic aroma compounds in zunyi black tea and their molecular mechanisms of interaction with olfactory receptors using molecular docking. *LWT*, *191*, 115640. https://doi.org/10.1016/j.lwt.2023.115640

Tan, Y., Wu, H., Shi, L., Barrow, C., Dunshea, F. R., & Suleria, H. A. R. (2023). Impacts of fermentation on the phenolic composition, antioxidant potential, and volatile compounds profile of commercially roasted coffee beans. *Fermentation*, *9*(10), 918. https://doi.org/10.3390/fermentation9100918

Tang, J., Li, Y., Zhang, L., Mu, J., Jiang, Y., Fu, H., Zhang, Y., Cui, H., Yu, X., & Ye, Z. (2023). Biosynthetic pathways and functions of indole-3-acetic acid in microorganisms. *Microorganisms*, *11*(8), 2077. https://doi.org/10.3390/microorganisms11082077

Tieman, D., Taylor, M., Schauer, N., Fernie, A. R., Hanson, A. D., & Klee, H. J. (2006). Tomato aromatic amino acid decarboxylases participate in synthesis of the flavor volatiles 2-phenylethanol and 2-phenylacetaldehyde. *Proceedings of the National Academy of Sciences*, *103*(21), 8287–8292. https://doi.org/10.1073/pnas.0602469103

Visciano, P., & Schirone, M. (2022). Update on biogenic amines in fermented and non-fermented beverages. *Foods*, *11*(3), 353. https://doi.org/10.3390/foods11030353

Vraneš, M., Borović, T. T., Drid, P., Trivić, T., Tomaš, R., & Janković, N. (2022). Influence of sodium salicylate on self-aggregation and caffeine solubility in water—A new hypothesis from experimental and computational data. *Pharmaceutics*, *14*(11), 2304. https://doi.org/10.3390/pharmaceutics14112304

Wang, B., Wang, S., Wang, Y., Zhang, S., Lin, X., Xu, X., Ji, C., Liang, H., & Dong, L. (2023). Deep exploration of lipid oxidation into flavor compounds: A density functional theory study on (E)-2-decenal thermal oxidative reaction. *Food Chemistry*, *428*, 136725. https://doi.org/10.1016/j.foodchem.2023.136725

Yang, J., Bai, W., Zeng, X., & Cui, C. (2019). Gamma glutamyl peptides: The food source, enzymatic synthesis, kokumi-active and the potential functional properties – a review. *Trends in Food Science & Technology*, *91*, 339–346. https://doi.org/10.1016/j.tifs.2019.07.022

Yeager, S. E., Batali, M. E., Guinard, J.-X., & Ristenpart, W. D. (2023). Acids in coffee: A review of sensory measurements and meta-analysis of chemical composition. *Critical Reviews in Food Science and Nutrition*, *63*(8), 1010–1036. https://doi.org/10.1080/10408398.2021.1957767

Zhang, Y., Yao, Y., Zhou, T., Zhang, F., Xia, X., Yu, J., Song, S., Hayat, K., Zhang, X., & Ho, C.-T. (2023). Light-colored maillard peptides: Formation from reduced fluorescent precursors of browning and enhancement of saltiness perception. *Journal of Agricultural and Food Chemistry*, *71*(50), 20251–20259. https://doi.org/10.1021/acs.jafc.3c07476
